# Muscle Cathepsin B Treatment Improves Behavioral and Neurogenic Deficits in a Mouse Model of Alzheimer's Disease

**DOI:** 10.1111/acel.70242

**Published:** 2025-10-05

**Authors:** Alejandro Pinto, Hazal Haytural, Cássio Morais Loss, Claudia Alvarez, Asude Ertas, Olivia Curtis, Alyssa R. Williams, Grayson Murphy, Kenneth J. Salleng, Sylvia Gografe, Nishant P. Visavadiya, Andy V. Khamoui, Ali Altıntaş, Tal Kafri, Romain Barres, Atul S. Deshmukh, Henriette van Praag

**Affiliations:** ^1^ Stiles‐Nicholson Brain Institute, Charles E. Schmidt College of Medicine Florida Atlantic University Jupiter Florida USA; ^2^ Department of Exercise Science and Health Promotion Florida Atlantic University Boca Raton Florida USA; ^3^ Novo Nordisk Foundation Center for Basic Metabolic Research University of Copenhagen Copenhagen Denmark; ^4^ Gene Therapy Center University of North Carolina at Chapel Hill Chapel Hill North Carolina USA; ^5^ Institut de Pharmacologie Moléculaire et Cellulaire Université Côte d'Azur & Centre National pour la Recherche Scientifique (CNRS) Valbonne France

**Keywords:** Alzheimer's disease, cathepsin B, memory, muscle, neurogenesis, proteomics

## Abstract

Increasing evidence indicates skeletal muscle function is associated with cognition. Muscle‐secreted protease Cathepsin B (Ctsb) is linked to memory in animals and humans, but has an unclear role in neurodegenerative diseases. To address this question, we utilized an AAV‐vector‐mediated approach to express Ctsb in skeletal muscle of APP/PS1 Alzheimer's disease (AD) model mice. Mice were treated with Ctsb at 4 months of age, followed by behavioral analyses 6 months thereafter. Here we show that muscle‐targeted Ctsb treatment results in long‐term improvements in motor coordination, memory function, and adult hippocampal neurogenesis, while plaque pathology and neuroinflammation remain unchanged. Additionally, in AD mice, Ctsb treatment normalizes hippocampal, muscle, and plasma proteomic profiles to resemble that of wildtype (WT) controls. In AD mice, Ctsb increases the abundance of hippocampal proteins involved in mRNA metabolism and protein synthesis, including those relevant to adult neurogenesis and memory function. Furthermore, Ctsb treatment enhances plasma metabolic and mitochondrial processes. In muscle, Ctsb treatment elevates protein translation in AD mice, whereas in WT mice mitochondrial proteins decrease. In WT mice, Ctsb treatment causes memory deficits and results in protein profiles across tissues that are comparable to AD control mice. Overall, the biological changes in the treatment groups are consistent with effects on memory function. Thus, skeletal muscle Ctsb application has potential as an AD therapeutic intervention.

## Graphical abstract

Targeting muscle with Cathepsin B (Ctsb) to treat the AD mouse brain. In this study, an AAV‐vector‐mediated approach, utilized to express Ctsb in muscle, prevented mnemonic and neurogenic deficits and normalized hippocampal, muscle, and plasma proteomic profiles.

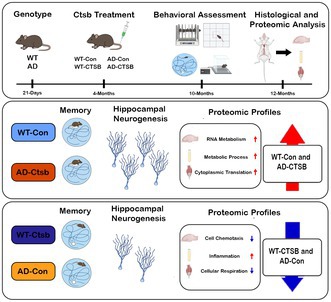

## 1 Introduction

Alzheimer's disease (AD) is a prevalent neurodegenerative disease, with minimal and largely ineffective pharmacological treatment options. Brain pathology includes amyloid plaques, tau protein aggregation, neuroinflammation, synaptic dysfunction, and neural tissue loss (DeTure and Dickson 2019). Lifestyle plays an important role in dementia incidence. Sedentary behavior and loss of muscle mass and strength (sarcopenia) are linked to shortened lifespan, hippocampal dysfunction, increased AD risk, and progression (Hunt et al. 2021; Oudbier et al. 2022). Conversely, physical activity enhances brain volume, connectivity, blood flow, cognition, and mood, and may delay or prevent the onset of the disease (Huuha et al. 2022). In rodents, running increases adult hippocampal neurogenesis, synaptic plasticity, neurotrophins, and memory, and reduces neuroinflammation and anxiety. Research into the underlying mechanisms indicates that peripheral factors produced during exercise can convey neurogenic and neuroprotective effects (Rai and Demontis 2022). Infusions of blood derived from young mice or exercising donors decrease neuroinflammation and enhance structural and functional neuroplasticity in aged animals. Factors secreted from muscle, liver, adipose tissue, and platelets into circulation may mediate such effects across species (Bieri et al. 2023; Rai and Demontis 2022) and hold promise as novel treatments for cognitive decline.

We identified Cathepsin B (Ctsb), a lysosomal cysteine protease, that has been primarily studied in pathological contexts—where it is linked to cancer progression, exacerbation of brain injury, and equivocally to AD (Mueller‐Steiner et al. 2006; Ni et al. 2022)—as a novel myokine (Moon et al. 2016). In humans, Ctsb is upregulated in circulation by exercise (Gaitan et al. 2021; Mazo et al. 2022; Moon et al. 2016) and electrical muscle stimulation (Nishikawa et al. 2024), and is associated with cognition (Gaitan et al. 2021; Moon et al. 2016). *Ctsb* ablation in mice precludes running‐induced pro‐neurogenic and mnemonic effects (Moon et al. 2016). Whether this is mediated by muscle *Ctsb* loss is unclear. Running (Moon et al. 2016) and muscle‐specific PGC‐1α overexpression (Wrann et al. 2013) increase muscle *Ctsb* levels (Peng et al. 2017). In addition, constitutive overexpression of transcription factor E‐B (TFEB), a master regulator of lysosomal function and cellular metabolism (Markby and Sakamoto 2020), enhances skeletal muscle *Ctsb* expression, reduces neuroinflammation, and improves cognition in aged mice (Matthews et al. 2023). Furthermore, in *Drosophila*, skeletal muscle transcriptional upregulation of proteases results in myokine release that is neuroprotective in the brain and retina (Rai et al. 2021; Rai and Demontis 2022). However, it remains to be determined whether muscle Ctsb treatment can prevent neurogenic and cognitive decline in neurodegenerative disease.

Here we explored the effects of skeletal muscle Ctsb expression in an AD mouse model, expressing chimeric mouse/human Swedish amyloid precursor protein (APP) and presenilin delta 9 mutations (APP/PS1), that cause neuropathological changes such as amyloid plaques, gliosis, and cognitive deficits (Jankowsky et al. 2004). In AD mice, Ctsb skeletal muscle expression prevented the appearance of motor, cognitive, and neurogenic deficits and normalized tissue and plasma proteomic profiles to levels of control WT mice. Our findings show that skeletal muscle Ctsb protects against memory decline in AD mice.

## 2 Materials and Methods

### 2.1 Mice and Housing Environment

B6.Cg‐Tg(APPswe,PSEN1dE9)85Dbo/Mmjax (APP/PS1) double transgenic hemizygous male mice on the C57BL/6;C3H background and female mice without the APP/PS1 allele (referred to here as wild‐type; WT) were purchased from Jackson Laboratories (MMRRC Strain #034829‐JAX). Mice were group housed in Individually Ventilated Cages (Tecniplast, Emerald line) containing bedding and nestlets and acclimated to the vivarium environment prior to breeding. Cages were maintained in a temperature‐controlled environment (21°C ± 2°C) and under a 12 h/12 h light–dark cycle (lights were switched off at 7:30 PM), with water and food available ad libitum.

All animal‐use procedures were conducted after approval by Florida Atlantic University's Institutional Animal Care and Use Committee, and they were conducted in accordance with the National Institutes of Health Guidelines for the care and use of Laboratory Animals.

### 2.2 Experimental Procedures

#### 2.2.1 Breeding Strategy and Allocation to the Groups

WT female and APP/PS1 hemizygous male mice (6–8 months old) were used as breeding pairs to produce APP/PS1 hemizygous mice (which were utilized as the AD model) and their WT littermates (serving as genotype controls). A 1:1 female‐to‐male ratio was employed as an in‐house breeding strategy. Litters were with their respective dams until weaning on postnatal day 20–21. The weaning procedure involved ear‐tagging the pups, collecting tail tips for genotyping (Transnetyx), and group housing the mice (3–4 mice per cage) according to sex. No more than two mice per genotype/litter were assigned to the same treatment group. Four‐month‐old male mice, derived from 2 cohorts of mice, were allocated to one of the following groups for either Ctsb or Control treatment: WT‐Con (*N* = 10 [6 + 4]), WT‐Ctsb (*N* = 9 [6 + 3]), AD‐Con (*N* = 8 [5 + 3]), and AD‐Ctsb (*N* = 14 [10 + 4]).

#### 2.2.2 Outline of Experiments

At 4 months of age, AD mice and their WT littermates were allocated to either Ctsb or Control treatment. Ctsb was expressed in muscle tissue by injecting an AAV vector into the tail vein to express the mouse *Ctsb* gene driven by the muscle creatine kinase promoter (MCK). One month thereafter, mice were injected with bromodeoxyuridine intraperitoneally to label dividing cells. Mice were left undisturbed until the onset of behavioral testing at 10 months of age (as detailed below). Upon conclusion of behavioral testing, mice were 11.5 months old. At 12 months of age, mice were deeply anesthetized for blood and tissue collection.

#### 2.2.3 AAV Vector

Four‐month‐old mice received tail vein injections of pAAV9‐tMCK‐mCTSB‐IRES‐eGFP or control vector pAAV9‐tMCK‐eGFP‐WPRE (VB5037) (Vector BioSystems). For tail vein injections, mice were lightly anesthetized within an induction chamber with isoflurane (Abbott) and subsequently placed onto a tail illuminator restrainer (Braintree Scientific). Once the ventral tail vein was dilated by the heat of the illuminator, 50 μL of vector (10^11^ vg/mL) was slowly injected into the blood vessel. Upon recovery from anesthesia (~10 min), mice were returned to their home cage.

### 2.3 Behavioral Tests

At 10 months of age, mice were tested in the activity box (open field) test, rotarod, Morris water maze, and fear conditioning paradigm. All tests were performed during the light period of the light–dark cycle.

#### 2.3.1 Activity Box (Open Field)

Tests were carried out in empty plexiglass arenas (height 20.3 cm, width 27.2 cm, depth 27.3 cm) containing two 16‐photo beam infrared (IR) arrays on the *X* and *Y* axes. Eight arenas were used simultaneously, each one located inside a sound‐attenuated chamber equipped with two ceiling white lights (SKU ENV‐221CL, Version 4.0, Med Associates, St. Albans, VT, USA). Mice were acclimated to the testing room for at least 45 min before starting the test. Mice were placed in the center of the arena and allowed to move freely for 60 min. Spontaneous locomotion (ambulatory distance) was recorded by Activity Monitor software (Med Associates).

#### 2.3.2 Rotarod

Motor function was evaluated with an accelerating rotarod for mice (Med Associates, St. Albans, VT) as described in the Data S1.

#### 2.3.3 Morris Water Maze

Spatial memory was tested in the Morris water maze. The apparatus consisted of a pool (1.83 m diameter) filled with water (24°C–26°C), made opaque with white nontoxic paint (Tempura, Crayola), and contained a platform (20 cm × 20 cm) that was hidden 1 cm below the water surface. The pool was surrounded by a black curtain, 60 cm from the edge, to which visual cues were attached. Indirect illumination of the pool and cues (65 lx at the center of the maze) was achieved by positioning 4 white lights equidistantly around the pool and facing the ceiling. The pool was separated into 4 virtual quadrants: northeast (NE), northwest (NW), southeast (SE), and southwest (SW). Mice were acclimated to the testing room daily for at least 30 min before starting the test. Mice were trained in four 60‐s trials with a 15‐s inter‐trial interval per day for 20 days, with a different starting point for each trial. The hidden platform was located in the NE quadrant. If the mouse found the platform, the trial was immediately terminated. Mice were placed on the platform if they did not find it within the allocated time. In either case, mice were allowed to remain on the platform for 15 s. Memory retention was evaluated in two 60‐s probe trials 4 and 24 h after the last acquisition session, in which the platform was removed. For probe trials, mice started in the SW quadrant. Data were collected by an automated video tracking system (Ethovision XT 17.5).

#### 2.3.4 Fear Conditioning Paradigm

Mice were tested within a four‐chamber system utilizing a near‐infrared video conditioning system (MED Associates Inc., Fairfax, VT, USA) that allows for testing of four animals simultaneously. Each chamber consisted of stainless‐steel walls with one transparent plexiglass door and a floor of parallel stainless‐steel rods connected to a shock generator. The chamber had an overhead white light (SKU ENV‐229M) to illuminate the inside (3 to 8 lx), and a speaker which was used to deliver a tone to serve as the conditioned stimulus—CS. Each of these chambers was located within a larger noise‐attenuating chamber (height 31.75 cm × width 71.12 cm × depth 59.69 cm), including a ventilation fan delivering background noise. A near‐infrared camera‐tracking system (MED Associates, Georgia, VT, USA) was used to automate the measurement of freezing behavior (Motion Threshold [au] = 20; Detection Method = Linear; Min Freeze Duration [f] = 18). The paradigm consisted of four phases: (i) Habituation, (ii) Conditioning, (iii) Tone‐cued, and (iv) Contextual phase, as described in the Data S1.

### 2.4 Euthanasia and Tissue Collection

Upon completion of the behavioral experiments, mice were deeply anesthetized for terminal blood and tissue collection. Approximately 300 μL of trunk blood was collected into EDTA‐coated tubes and directly placed on ice. Within 15 min, these samples were spun (2000 RCF) for 15 min within a temperature‐controlled (4°C) centrifuge. Supernatant was collected and 2–3 aliquots (50–75 μL) were stored at −80°C until further use. Following transcardiac perfusion with 0.9% saline (room temperature), liver, gastrocnemius, soleus, biceps, and heart tissue, as well as prefrontal cortex and hippocampus from one hemisphere of the brain, were dissected, immediately flash‐frozen in liquid nitrogen, placed on dry ice, and stored at −80°C. For the present study, hippocampus, gastrocnemius, and plasma were utilized for proteomic analyses. The other brain hemisphere was placed in ice‐cold 4% paraformaldehyde, post‐fixed for 72 h, and then equilibrated in 30% sucrose until sectioning.

#### 2.4.1 Time‐Course and Muscle Ctsb Expression

Additional cohorts of mice were utilized to monitor Ctsb levels with aging, and at shorter intervals after vector injection, as described in the Data S1. Mice were euthanized and tissues collected as described above.

### 2.5 Histology

Sequential coronal brain tissue sections (40 μm) were taken using a freezing microtome (HM450, ThermoFisher Scientific, Waltham, MA, USA) throughout the rostrocaudal extent of one hemisphere of the brain and stored in 96‐well plates in a phosphate‐buffered glycerol anti‐freezing solution at −20°C. DCX, BrdU, Thioflavin‐S, GFAP, and Iba1 staining, image analysis, and quantification are described in the Data S1.

### 2.6 Proteomics

#### 2.6.1 Sample Preparation for Proteomics Analysis

Hippocampus and gastrocnemius muscle tissues were powdered and lysed in 4% Sodium Dodecyl Sulfate (SDS) buffer (100 mM Tris, pH 8.5) using an Ultra Turrax blender (IKA). Lysates were then boiled at 95°C for 10 min, followed by 10 min of sonication in an ultrasonic bath sonicator. After centrifugation at 16,000 *g* for 10 min, supernatants were transferred into a new tube for the determination of protein concentration using the DC protein assay (Thermo Fisher Scientific). Subsequently, 20 μg of proteins were reduced by the addition of 100 mM of dithiothreitol and alkylated by 40 mM of chloroacetamide. After 45 min of incubation at room temperature, proteins were digested using the PAC protocol on a KingFischer Flex robot (Batth et al. 2019). In brief, a 1:4 protein‐to‐bead ratio was added to the sample lysate, and protein aggregation was induced by dispensing a final concentration of 70% acetonitrile (ACN), followed by a 10‐min incubation without agitation. Then, the sample plates were placed in a magnetic stand (Dynamag Thermo), and beads with protein aggregates were first washed twice with 100% ACN and then twice with 70% ethanol. After discarding the last wash, beads were resuspended in 100 μL of digestion buffer (50 mM Tris–HCl, pH 8.5) containing a 1:100 enzyme‐to‐protein ratio of trypsin and a 1:500 enzyme‐to‐protein ratio of LysC (Wako), and enzymatic digestion was done at 37°C overnight. The next day, digestion was quenched by the addition of a final concentration of 1% trifluoroacetic acid (TFA) in isopropanol. Peptides were then cleaned of salts and remaining lipids using styrenedivinylbenzene–reverse phase sulfonate (SDB‐RPS, details) stage‐tips and eluted in 60 μL of 1% ammonia and 80% ACN. Using a speedvac, peptides were dried completely and resuspended in 10 μL of 0.1% TFA and 5% ACN. Peptide concentration was determined by a NanoDrop spectrophotometer (Thermo Fisher Scientific), and a total of 200 ng of peptides were loaded on Evotip C18 trap columns (Evosep Biosystems) according to the manufacturer's instructions.

For the plasma proteomics, 1 μL of plasma was directly lysed in 100 μL of 100 mM Tris buffer (pH 8.5). Lysates were then boiled at 95°C for 10 min, followed by 10 min sonication in an ultrasonic bath sonicator. After a quick spin down, the enzyme mix (containing a 1:100 enzyme to protein ratio of trypsin and a 1:500 enzyme to protein ratio of LysC) was added, and enzymatic digestion was done at 37°C overnight. The next day, digestion was quenched by the addition of a final concentration of 2% TFA. Peptide concentration was then determined by a NanoDrop spectrophotometer (Thermo Fisher Scientific), and a total of 350 ng of peptides was loaded on Evotip C18 trap columns (Evosep Biosystems) according to the manufacturer's instructions. Liquid chromatography‐mass spectrometry (LC–MS/MS) and differential proteome analysis are described in the Data S1.

### 2.7 Western Blotting

Western blotting (WB) of muscle and hippocampus was performed, as described in the Data S1.

### 2.8 Statistical Analysis

Statistical analyses were performed as described (Loss et al. 2021). All behavioral and histological data were analyzed in RStudio (Version 1.4.1717, 2009–2021 RStudio, PBC) R version 4.1.0 (2021‐05‐18) using either Generalized Linear Model (GLM) or Generalized Linear Mixed Model (GLMM) as described in the Data S1.

### 2.9 Bias‐Reducing Measures

Randomization, blinding, and systematic sampling procedures were applied to minimize both performance and detection bias (Loss et al. 2021) as described in the Data S1.

## 3 Results

**FIGURE 1:**
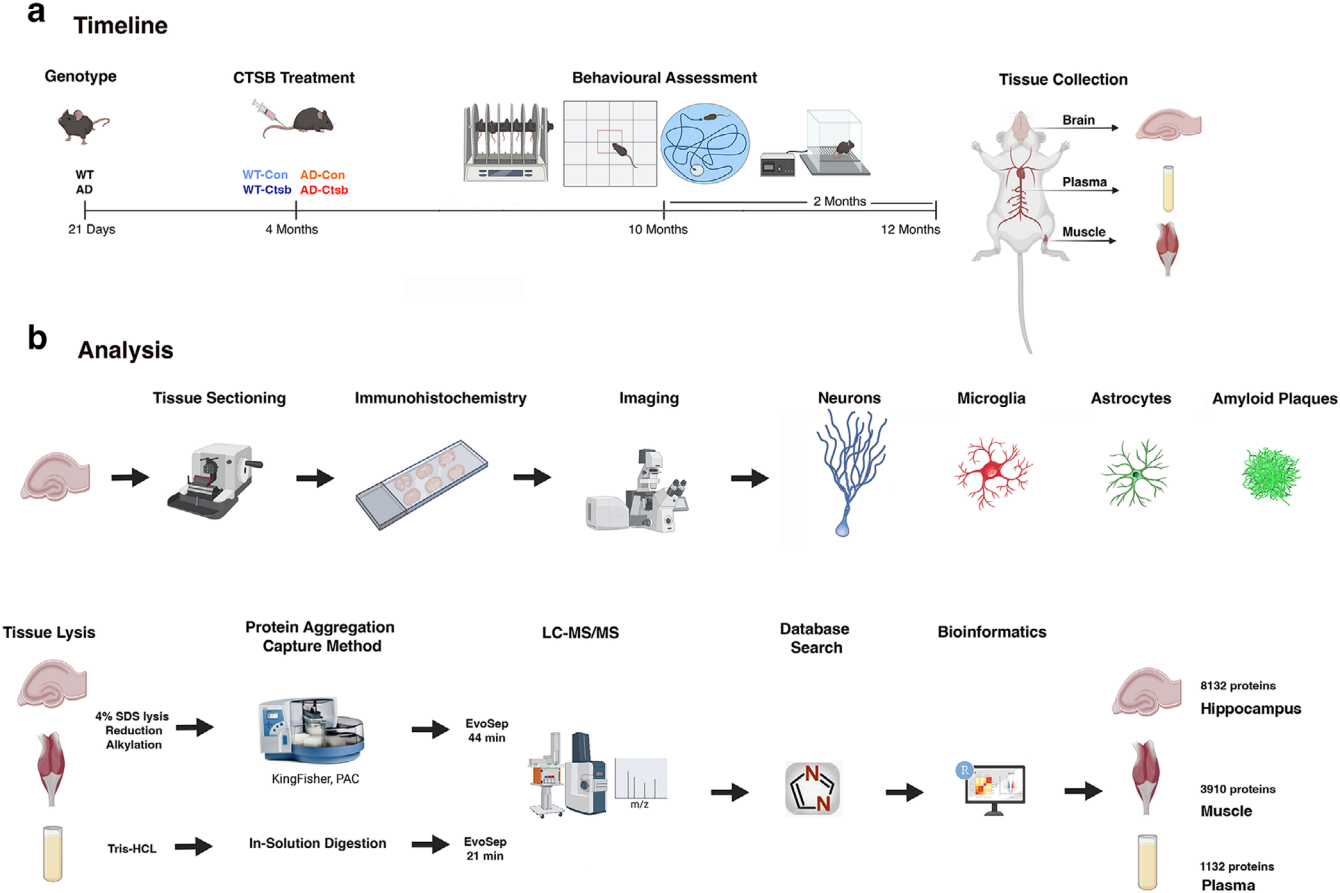
Experimental design and timeline. (a) Four‐month‐old male mice (either AD or WT mice) were injected into the tail vein with Ctsb (pAAV9‐tMCK‐mCTSB‐IRES‐eGFP) or Control (Con) vector (pAAV9‐tMCK‐IRES‐eGFP): WT‐Con (*N* = 10), WT‐Ctsb (*N* = 9), AD‐Con (*N* = 8), and AD‐Ctsb (*N* = 14). Mice were subjected to a battery of behavioral tests from 10 months of age. The behavioral assessment consisted of activity box (open field), rotarod, Morris water maze, and fear conditioning paradigm. At 12 months of age, mice were deeply anesthetized and euthanized for tissue and plasma collection. (b) One hemisphere of the brain was sectioned and used for histological evaluation of adult neurogenesis, amyloid beta plaque deposition, and neuroinflammation. Hippocampus from the other brain hemisphere, gastrocnemius muscle, and plasma were used for proteomic assays. Briefly, hippocampal and muscle tissues were lysed in 4% SDS buffer, followed by reduction and alkylation steps, and protein aggregation capture method. Cleaned peptides were injected into LC–MS/MS (Evosep HPLC 44 min coupled to timsTOF Pro 2 mass spectrometer). Plasma proteins were solubilized directly in Tris buffer. Cleaned peptides were injected into LC–MS/MS (Evosep HPLC 21 min coupled to timsTOF Pro 2 mass spectrometer).

Four‐month‐old male mice (AD and WT mice) received AAV‐vector‐mediated Ctsb or Control (Con) treatment: WT‐Con, WT‐Ctsb, AD‐Con, and AD‐Ctsb. Six months later, mice underwent a battery of behavioral tests, consisting of activity box (open field), rotarod, Morris water maze, and fear conditioning (Figure 1a). Immunohistochemical analysis of adult‐born neurons, markers of neuroinflammation (microglia and astrocytes), and AD‐related pathology, was assessed in the hippocampus and cortex. Furthermore, hippocampus, gastrocnemius muscle, and plasma proteome were analyzed by liquid chromatography‐mass spectrometry (LC–MS/MS) (Figure 1b).

### 3.1 Locomotor Behavior Is Modified by Ctsb Treatment

In order to assess the impact of AAV‐vector‐mediated skeletal muscle expression of Ctsb on locomotor activity and motor coordination, mice were tested within the activity box (open field) and rotarod apparatus.

#### 3.1.1 Activity Box and Rotarod

**FIGURE 2:**
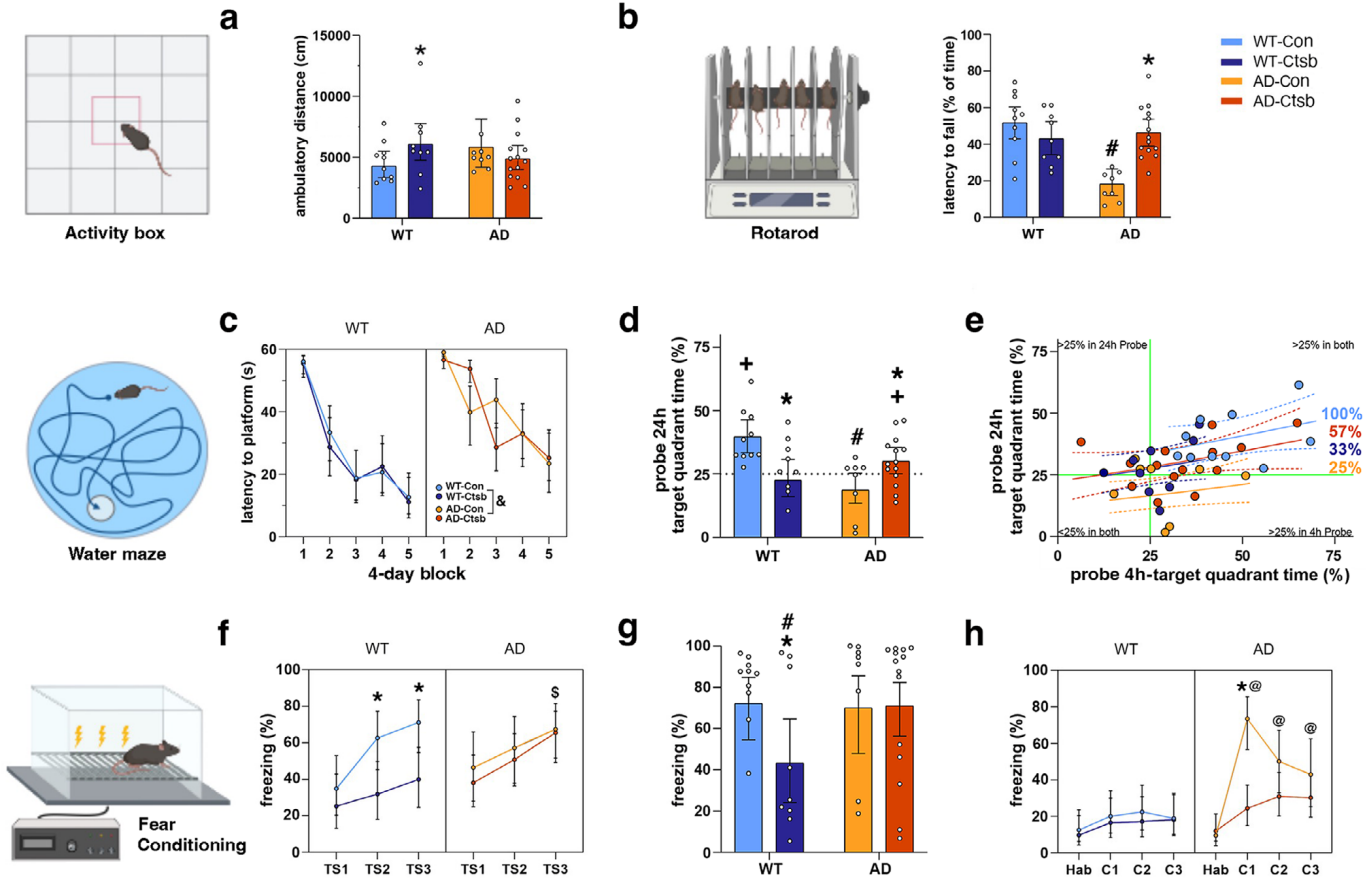
Ctsb treatment differentially affects locomotion, motor coordination and memory function in WT and AD mice. (a) Ctsb treatment increased distance traveled in WT mice during a 60 min activity box session. (b) AD‐Con mice had a shorter latency to fall than all the other groups on an accelerating rotarod (4–40 rpm). (c) Acquisition curves in the Morris water maze, analyzed over 4‐day bins (during a 20‐day training protocol). WT mice perform better than AD mice throughout the acquisition phase. (d, e) Morris water maze probe trials to test retention of spatial memory. (d) AD‐Con mice and WT‐Ctsb mice had impaired retention of spatial memory performance during the 24 h probe trial. Only WT‐Con and AD‐Ctsb mice preferred the platform quadrant compared to 25% chance. (e) Considering the performance in the 4 h and 24 h probe trials as a whole, WT‐Con (100%), AD‐Ctsb (57%), AD‐Con (25%) and WT‐Ctsb (33%) mice spent more than 25% of time in the target quadrant in both trials. Solid green lines represent 25% chance. (f) In the fear conditioning paradigm WT‐Ctsb mice froze less during the conditioning session and (g) the tone‐cued phase, as compared to the other groups. (h) AD‐Con mice displayed increased CFR to context as compared to the other groups. (*N*; WT‐Con = 10, WT‐Ctsb = 9, AD‐Con = 8, AD‐Ctsb = 14). ^#^ *p* < 0.05 ad compared to WT in the same Treatment. *p < 0.05 Ctsb compared to Con in the same Genotype. ^+^ *p* < 0.05 of being equal or lesser than 25% chance (one‐tailed Wilcoxon signed rank exact test). ^&^ *p* < 0.05 AD compared to WT (Genotype main effect). ^$^ *p* < 0.05 ad‐Ctsb compared to WT‐Ctsb. ^@^ *p* < 0.05 ad‐Con compared to WT‐Con. Data were analyzed by GLM (a, b, d, g) or GLMM (c, f, h) and are presented as Estimated Marginal Means and their respective 95% CI. In (e), solid lines represent the Estimated Marginal Means while dashed lines represent 95% CI.

Mice were placed in the activity box (open field) for 60 min. *Ctsb* expression increased the total ambulatory distance in WT (WT‐Ctsb) but not in AD mice (Figure 2a). Analysis of distance traveled over time showed similar habituation to the arena between groups (Figure S1; Tables S1 and S2). In the rotarod, the AD‐Con group had a shorter latency to fall as compared to the WT‐Con and AD‐Ctsb groups. *Ctsb* expression reversed the motor coordination deficit in AD animals (Figure 2b; Table S3).

### 3.2 Learning and Memory Is Differentially Affected by Ctsb in AD Versus WT Mice

#### 3.2.1 Morris Water Maze

To assess spatial learning, mice were trained in the Morris water maze with 4 trials per day for 20 days. In the acquisition phase, the WT groups displayed a shorter latency to the platform than the AD groups (4‐day bins over time, Figure 2c, and Table S4). For all groups, mean latency to the platform was < 20 s in the last training session. Swim speed did not differ between the groups on day 1 and did not change over time (Figure S2, Tables S5 and S6).

To test memory retention, the platform was removed and probe trials were conducted 4 h (Figure S2) and 24 h after the last training session (Tables S7 and S8). In the 24 h probe trial, the WT‐Con and AD‐Ctsb groups spent more time in the target quadrant than the AD‐Con group. In addition, WT‐Ctsb mice were in the target quadrant for a shorter time than WT‐Con mice. Both the WT‐Con and AD‐Ctsb groups preferred the target quadrant above 25% chance (Figure 2d, Tables S9 and S10), indicating that these groups remember where the platform was located during the acquisition phase. Examining the performance in the 4 h and 24 h probe trials as a whole, we found that 100% WT‐Con, 57% AD‐Ctsb, 33% WT‐Ctsb, and 25% AD‐Con mice spent more than 25% of their time in the target quadrant in both trials (Figure 2e). Altogether, these results indicate that the intervention benefits retention of spatial memory in AD mice, whereas it has a detrimental outcome in WT mice.

#### 3.2.2 Fear Conditioning

Mice were subjected to a Pavlovian conditioning‐based approach to assess fear learning and memory by measuring the degree of freezing, a Conditioned Fear Reaction (CFR), elicited by pairing a tone with a shock (Figure 2f–h). In the habituation (Hab) session on Day 1, there were no differences in freezing between the groups (WT‐Con = 12.66%, 6.35–23.65; WT‐Ctsb = 9.80%, 4.37–20.50; AD‐Con = 9.61%, 4.02–21.29; AD‐Ctsb = 12.12%, 6.52–21.42). On Day 2, the Conditioning Phase consisted of three tone‐shock (TS) pairings. The WT‐Con, AD‐Con, and AD‐Ctsb mice presented higher CFR than WT‐Ctsb mice. Indeed, WT‐Ctsb mice (< 40%) freeze less than all the other groups (> 65%) in Tone‐Shock 3 (TS3), the last conditioning stimulus (CS)—unconditioned stimulus (US) presentation (Figure 2f; Table S11). On Day 3, mice were first tested for tone‐cued memory session in a new contextual environment (T1–T3). The WT‐Ctsb group exhibited a reduced average tone‐elicited CFR (< 44% freezing) compared to the other groups (> 70% freezing) (Figure 2g; Table S12). 1 h after the tone‐cued session on Day 3 (C1), as well as on Days 5 and 7 (C2 and C3, respectively), mice were tested in the original environment used in the habituation (Hab) and conditioning sessions. Elevated context‐elicited CFR was observed in AD‐Con only (Figure 2h; Table S13). Overall, fear conditioning behavior in AD‐Ctsb mice was similar to that of the WT‐Con group (Figure 2f–h).

### 3.3 Adult Hippocampal Neurogenesis Is Enhanced by Ctsb in AD Mice

**FIGURE 3:**
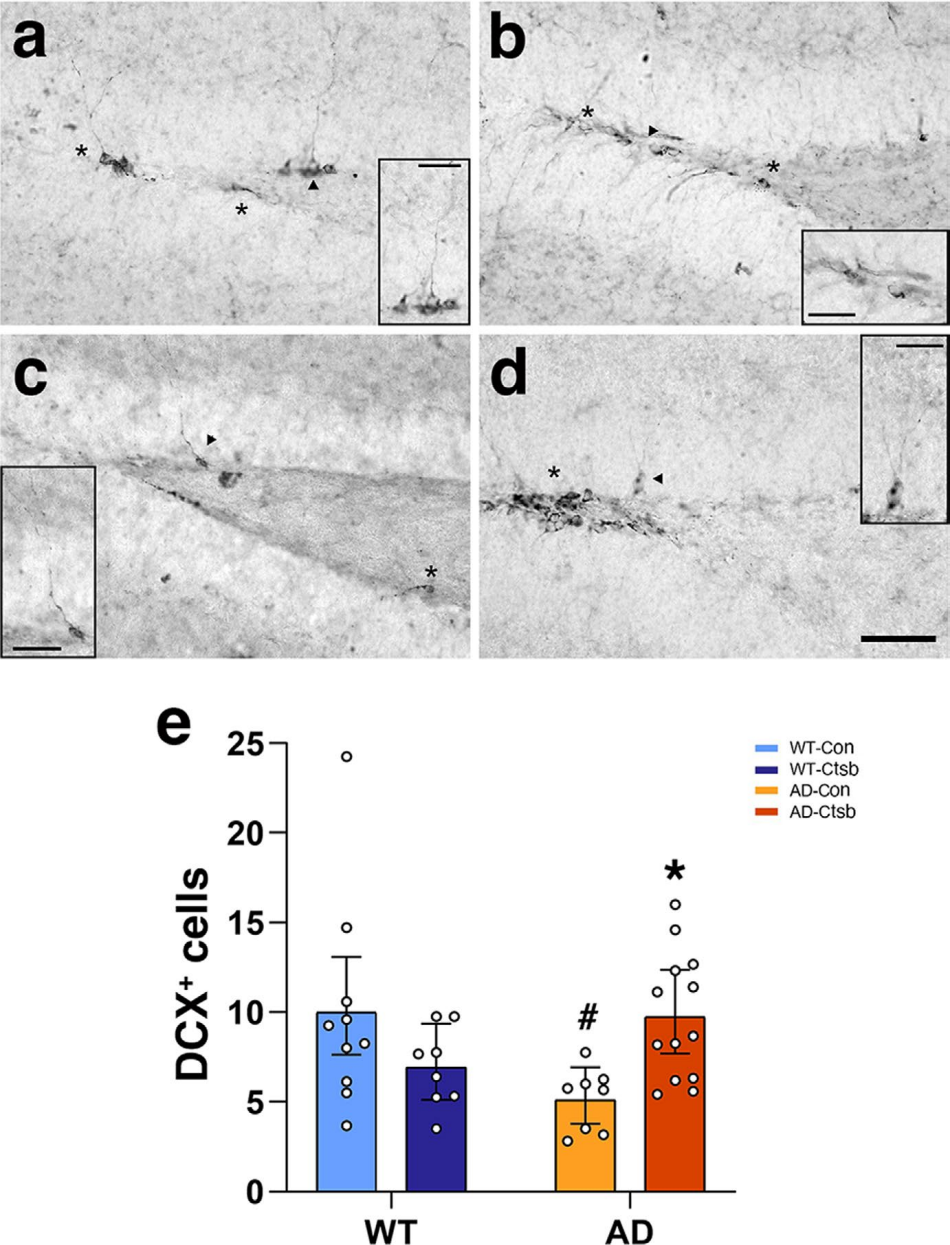
Ctsb treatment improves adult neurogenesis in AD mice. (a–d) Representative photomicrographs of the dentate gyrus derived from (a) WT‐Con; (b) WT‐Ctsb; (c) AD‐Con; (d) AD‐Ctsb brain tissue sections subjected to DCX staining. (e) Treatment with Ctsb in AD mice restored DCX cell numbers to WT‐Con levels. (*N*; WT‐Con = 10, WT‐Ctsb = 8, AD‐Con = 8, AD‐Ctsb = 13). Scale bars: 50 μm overview; 25 μm inset. ^#^ *p* < 0.05 AD compared to WT in the same Treatment. *p < 0.05 Ctsb compared to Con in the same Genotype. Data in (e) was analyzed by GLM and are presented as Estimated Marginal Means and their respective 95% CI.

To assess adult hippocampal neurogenesis at 12 months of age, we quantified doublecortin (DCX)^+^ cells (Figure 3a–d). Analysis showed an interaction between genotype and treatment. Specifically, DCX^+^ cell number in AD‐Con was reduced by 50% as compared to the WT‐Con group. Treatment with Ctsb restored DCX^+^ cell number to WT‐Con levels in AD mice (Figure 3e, Table S14). Cell genesis at 5 months of age did not differ between groups (Figure S3, Tables S15, S16). Our results indicate that AAV‐mediated Ctsb expression in skeletal muscle prevented the adult hippocampal neurogenesis deficit caused by AD.

### 3.4 Plaque Deposition and Microglial Activation in AD Mice

To evaluate AD pathology, sections derived from AD‐Con and AD‐Ctsb mice were stained with Thio‐S to label amyloid deposits (Figure S4a,b). Plaque density (Figure S4c,e) and counts (Figure S4d,f) in AD cortex and hippocampus did not differ between groups (Tables S17–S20).

**FIGURE 4:**
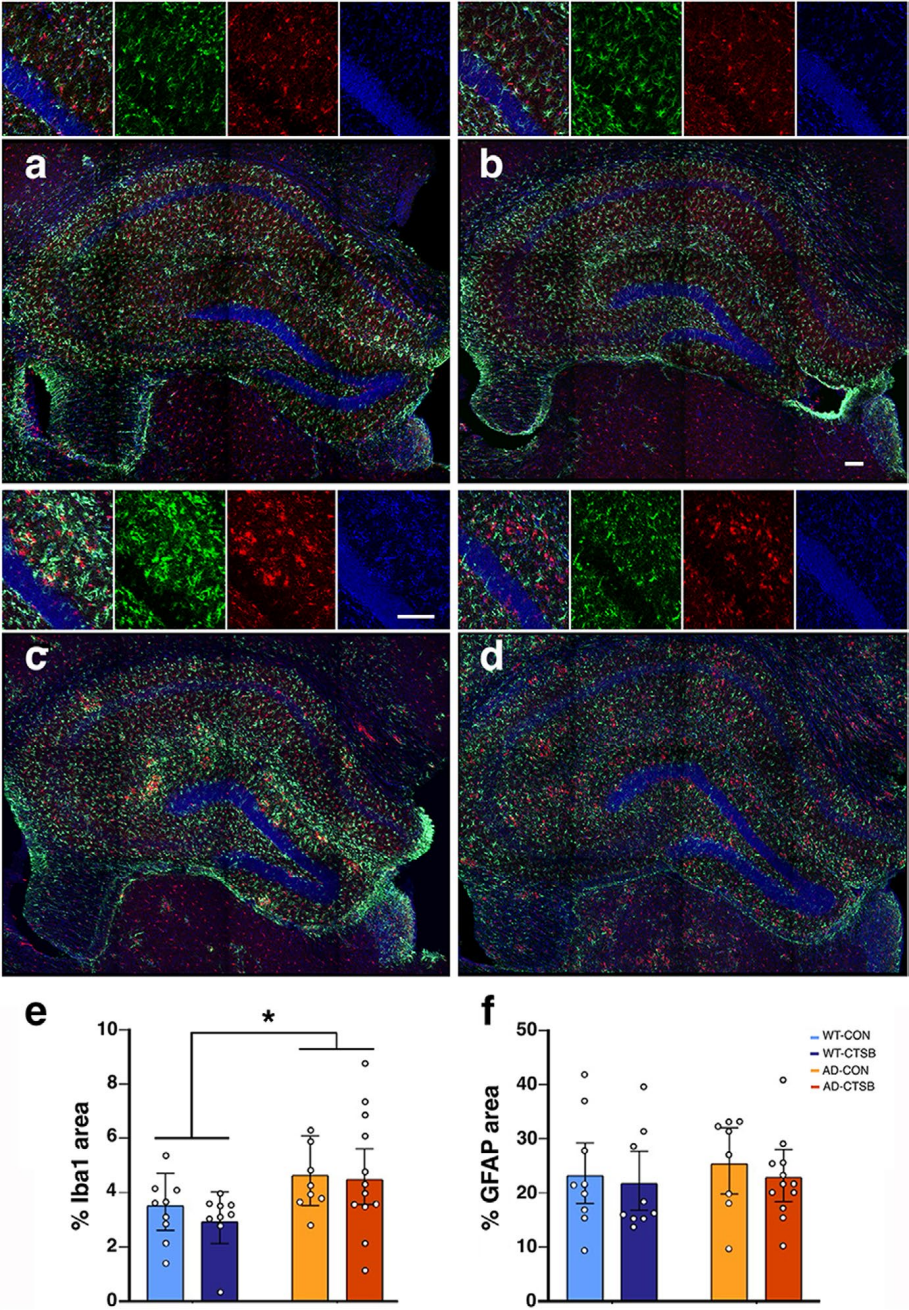
Ctsb treatment does not modify neuroinflammation in AD mice. Representative photomicrographs of dorsal hippocampal sections subjected to immunofluorescent double‐labeling for GFAP (green) and Iba1 (red), derived from (a) WT‐Con, (b) WT‐Ctsb, (c) AD‐Con and (d) AD‐Ctsb mice. Nuclei were stained with 4′,6‐diamidino‐2‐phenylindole (DAPI) blue. (e, f) Astrocyte and microglia area density. Increased hippocampal density of (e) Iba1^+^ microglia but not of (f) GFAP^+^ astrocytes was observed in the AD groups. (N; WT‐Con = 9, WT‐Ctsb = 9, AD‐Con = 8, AD‐Ctsb = 12). Scale bars: 100 μm. *p < 0.05 for Genotype effect (WT vs. AD). Data were analyzed by GLM and are presented as Estimated Marginal Means and their respective 95% CI.

To quantify microglia and astrocyte density, sections derived from the WT and AD groups were stained for Iba1 and GFAP, respectively (Figure 4a–d). Our analysis revealed a genotype, but not a treatment effect on Iba1 density. There was an increase in Iba1 (Figure 4e; Table S21) in AD‐Con and AD‐Ctsb as compared to WT‐Con and WT‐Ctsb groups. There was no group difference in GFAP density (Figure 4f; Table S22). Thus, our results show that Ctsb expression in AD mouse skeletal muscle does not affect plaque density or microglial activation.

### 3.5 Possible Mechanisms Mediating Ctsb Treatment Effects: Proteomic Analyses

To gain better insight into the mechanisms driving the effects of Ctsb treatment in AD on behavior and adult neurogenesis, proteomic analyses of hippocampus, muscle, and plasma were performed (Figure 1b).

### 3.6 Ctsb Treatment Enhanced Transcription and Translation in AD Hippocampus

To investigate the potential effects of muscle‐specific Ctsb treatment on the hippocampus, we conducted proteomic analysis on hippocampal tissue derived from Ctsb‐ and Con‐treated AD and WT mice. Our workflow (Figure 1b) enabled the quantification of 8132 proteins, with 7509 proteins reliably quantified in more than three samples per experimental group (Figure S5b). Protein abundance ranged over approximately four orders of magnitude, encompassing highly abundant neuronal (e.g., App, Bdnf, Ckb, Thy1), cytoskeletal (e.g., Acta1, Actg2, Tuba1c), and glial (e.g., Gfap and Apoe) proteins (Figure S5c). Multidimensional scaling showed no clear clustering pattern by genotype or treatment (Figure S5d).

**FIGURE 5:**
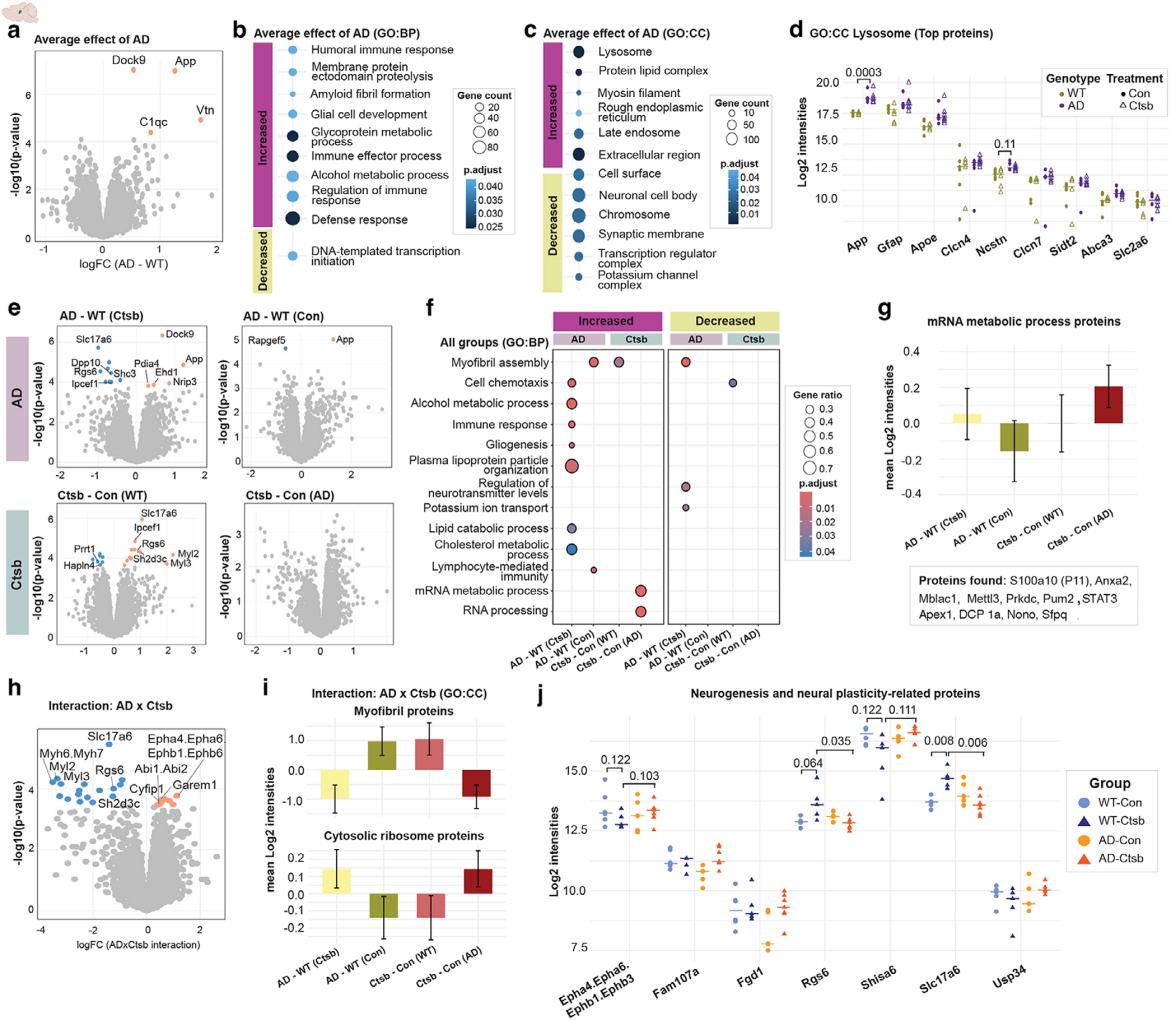
Ctsb treatment induces cytoskeletal reorganization in WT mice, whereas transcription and RNA processing in AD mice. (a) Volcano plot displaying average effect of AD. Gene set enrichment analysis (GSEA) based on (b) biological processes (BP) and (c) cellular components (CC). (d) Abundance of proteins associated with the lysosome ontology that have log fold changes > 0.50. (e) Volcano plots showing significant proteins, if present, in each pairwise group comparison. (f) GSEA of biological processes summarizing changes observed in all groups. (g) Mean abundance of proteins associated with mRNA metabolic process ontology. (h) Volcano plot of interaction between AD and Ctsb treatment. (i) Mean abundance of proteins associated with myofibril and cytosolic ribosome ontologies, which were found to be enriched by GSEA of interaction data using cellular components. (j) Abundance of selected proteins involved in neurogenesis and neural plasticity. Multiple hypothesis testing was performed by the Benjamini‐Hochberg method for each differential expression analysis, and proteins with FDR < 0.10 were considered statistically significant. For GSEAs, a cut‐off of FDR < 0.05 was used. Data for the pairwise comparisons are annotated as follows: AD‐WT(Ctsb), *AD‐Ctsb* vs *WT‐Ctsb*; AD‐WT(Con), *AD‐Con* vs *WT‐Con*; Ctsb‐Con(WT), *WT‐Ctsb* vs *WT‐Con*; Ctsb‐Con(AD), *AD‐Ctsb* vs *AD‐Con*.

To gain insight into the changes driven by AD pathogenesis, we first performed differential abundance analysis of the average effect of AD. Only four proteins were significantly upregulated in AD, with a false discovery rate cut‐off of 0.10 (Figure 5a): Amyloid precursor protein (App), vitronectin (Vtn), dedicator of cytokinesis protein 9 (Dock9), and complement C1q, subcomponent subunit C (C1qc) (Table S23). These proteins are well‐known to be dysregulated in AD pathogenesis (Hong et al. 2016; Jankowsky et al. 2004). Our findings align well with the AD mouse model utilized in this study, which overexpresses App, resulting in increased levels of APP and its peptide, amyloid beta‐peptide 42, deposited in amyloid plaques (Jankowsky et al. 2004). Gene set enrichment analysis (GSEA) revealed that biological processes related to immune response, glycoprotein metabolic processes, amyloid fibril formation, and glial cell development were significantly increased in AD mice (Figure 5b, Table S24). In contrast, DNA‐templated transcription initiation was decreased (Figure 5b). GSEA based on cellular components showed that protein‐lipid complex, lysosome, and late endosome were significantly increased in AD, while synaptic membrane, potassium channel, and transcription regulator complexes were decreased (Figure 5c). Some of the proteins associated with lysosome included the classical markers of AD, such as App, glial fibrillary acidic protein (Gfap), apolipoprotein E (Apoe), and nicastrin (Ncstn) (Figure 5d).

Next, we performed differential abundance analyses between each group (Table S23). The impact of AD pathogenesis was assessed by comparing AD‐Con vs. WT‐Con [AD‐WT(Con)] and AD‐Ctsb vs. WT‐Ctsb [AD‐WT(Ctsb)] groups, while WT‐Ctsb vs. WT‐Con [Ctsb‐Con(WT)] and AD‐Ctsb vs. AD‐Con [Ctsb‐Con(AD)] group comparisons were used to assess the effect of Ctsb treatment (Figures 5e,f). Significantly regulated proteins for each group comparison, if present, are shown in Figure 5e (FDR < 0.10). Although amyloid burden was not reduced by Ctsb treatment, our proteomics data revealed a downward trend of App and App.Aplp2 protein levels in the AD‐Ctsb group compared to AD‐Con. Additionally, changes in the expression of key secretases involved in App processing suggest that Ctsb may promote a shift toward non‐amyloidogenic App processing mediated by α‐secretase (such as ADAM10 and ADAM17) in the AD model (Figure S5e). GSEAs showed that most changes were specific to the AD‐Ctsb vs. WT‐Ctsb group, indicating a strong phenotype of the disease model, consistent with the behavioral findings where Ctsb treatment has opposite effects in WT and AD mice (Figure 5f, Table S24). Enrichment of ontologies such as increased immune response, gliogenesis, and alcohol metabolic process in AD‐WT(Ctsb) recapitulated the average effect of AD as shown in Figure 5b. However, several processes showed opposing regulation; myofibril assembly increased in Ctsb‐Con(WT) and AD‐WT(Con) groups but decreased in AD‐WT(Ctsb). In addition, cell chemotaxis was diminished in AD‐WT(Ctsb) but elevated in Ctsb‐Con(WT) mice. Moreover, Ctsb treatment increased mRNA metabolic process and RNA processing in the AD group compared to AD‐Con (Figure 5f). Cellular component analysis showed a similar pattern of subcellular alterations (Figure S5f). A closer look at the mRNA metabolic process revealed that abundance of proteins associated with this ontology increased, especially in the AD‐Ctsb vs. AD‐Con group (Figure 5g). Some of the proteins involved were S100a10 (also known as P11) and binding partner annexin A2 (Anxa2), for which immunoblotting confirmed the proteomics observation (Figure S5g).

**FIGURE 6:**
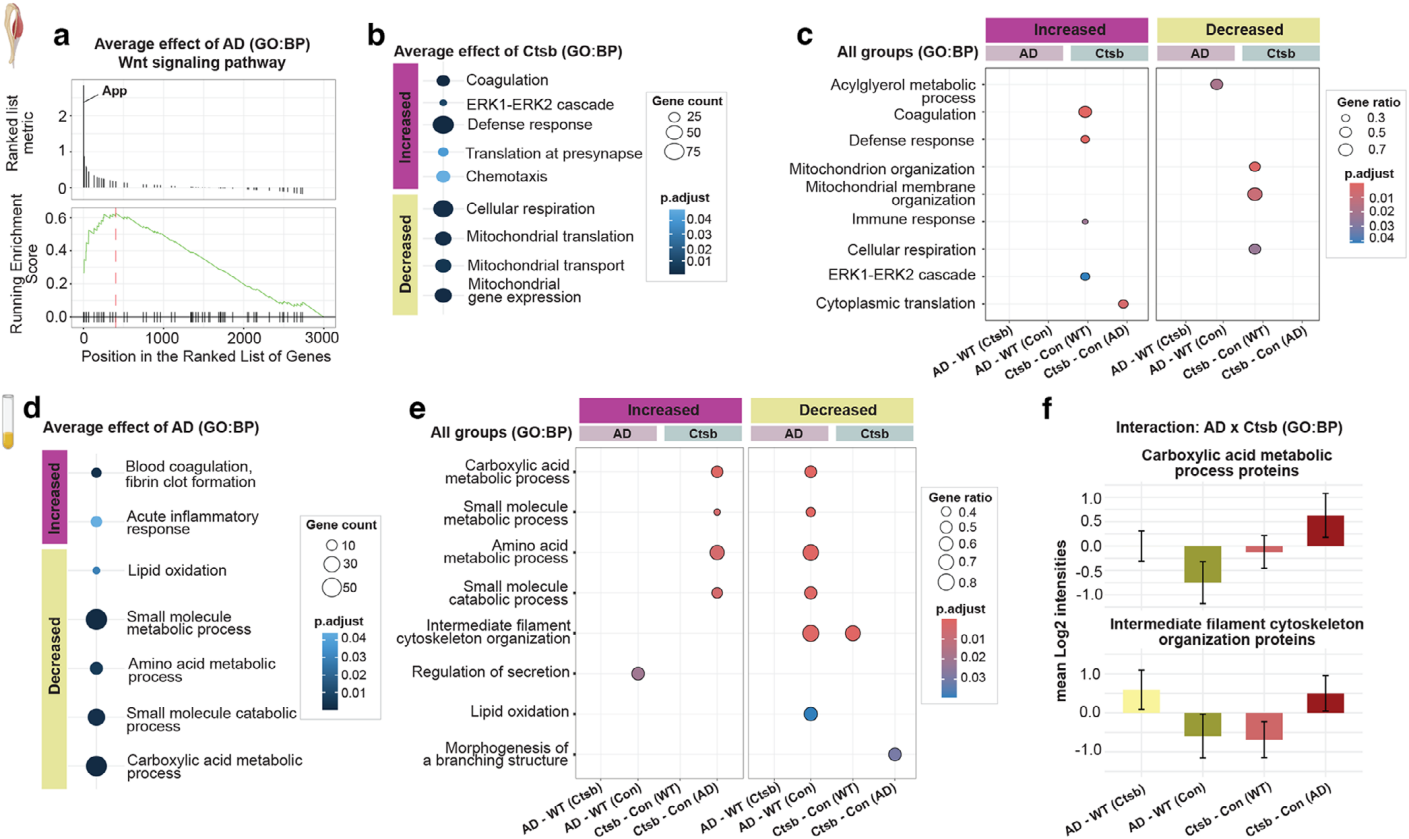
Effects of Ctsb treatment on muscle and plasma proteome. Muscle proteome: (a) GSEA plot displaying Wnt signaling pathway as one of the most affected gene sets in AD. GSEA of average effect of Ctsb treatment based on (b) biological processes (BP). (c) GSEA using biological processes summarizing changes observed in each group. Plasma proteome: GSEA of (d) biological processes summarizing changes observed in the average effect of AD. (e) GSEA of biological processes summarizing changes observed in all groups. (f) Mean abundance of proteins involved in metabolic processes and cytoskeletal organization, selected from GSEA of interaction data. (c, e, f) Data for the pairwise comparisons are annotated as follows: AD‐WT(Ctsb), *AD‐Ctsb* vs *WT‐Ctsb*; AD‐WT(Con), *AD‐Con* vs *WT‐Con*; Ctsb‐Con(WT), *WT‐Ctsb* vs *WT‐Con*; Ctsb‐Con(AD), *AD‐Ctsb* vs *AD‐Con*.

Lastly, the molecular effects of Ctsb treatment in AD were assessed by the interaction analysis between AD and Ctsb, which revealed 17 decreased and eight increased proteins (FDR < 0.10) (Figure 5h, Table S23). GSEA showed that proteins associated with myofibril and actin cytoskeleton were significantly decreased while those involved in the cytosolic ribosome were increased (Table S24). Notably, the mean abundance of proteins within these ontologies clearly showed that AD‐Con and WT‐Ctsb groups had similar overall protein abundance, relative to WT‐Con, thereby indicating a potential shared molecular profile (Figure 5i). In contrast, the AD‐Ctsb group showed a reduction in myofibril‐associated proteins and an increase in cytosolic ribosomal proteins, suggesting a partial compensation for cytoskeletal impairment and disrupted translation. It is noteworthy that, following Ctsb treatment, proteins typically found in muscle, such as myosin light chain (Myl2 and Myl3), were decreased in the hippocampus of AD mice but upregulated in the hippocampus of WT mice (Figure 5e,i). This aligns with earlier observations where the abundance of muscle proteins increased in the brain during aging and in pathological conditions. For instance, in human AD and aging brain, elevated muscle proteins including dysferlin (Galvin et al. 2006), Myl9 (Xu et al. 2016), and tropomyosin alpha‐1 chain (Tpm1) (Castano et al. 2013) were observed. The reduction of myofibril proteins in AD‐Ctsb mice may therefore reflect a reversal of pathological changes linked to disease progression.

Interestingly, several proteins that are involved in neurogenesis and neural plasticity were regulated similarly in AD‐Ctsb and WT‐Con, as compared to WT‐Ctsb and AD‐Con groups (Figure 5j), including Slc17a6, vesicular glutamate transporter 2 (VGLUT2), (Liguz‐Lecznar and Skangiel‐Kramska 2007), which was further substantiated by immunoblotting (Figure S5h). Mounting evidence points to an excitotoxic milieu in AD, with a disrupted balance between excitatory and inhibitory signaling (Muller et al. 2021). In our data, Ctsb treatment increases glutamatergic protein intensities in WT but decreases them in AD (Figure S5i). This bidirectional effect suggests a compensatory dampening of excitatory drive in the AD‐Ctsb mice, potentially counteracting excitotoxic stress.

### 3.7 Ctsb Treatment Decreased WT Muscle Mitochondrial Processes, Whereas It Increased Translation in AD Muscle

To characterize the effect of muscle‐specific Ctsb treatment, we performed muscle proteomics and quantified 2998 proteins after stringent filtering (Figure S6a). Classical muscle‐enriched proteins were marked high on the abundance rank plot (Figure S6b). Multidimensional scaling plots did not exhibit any clustering patterns (Figure S6c).

Differential abundance analyses between groups did not reveal significant changes in proteins with the cut‐off of FDR < 0.10 (Table S25). Muscle Ctsb protein abundance did not differ between groups (Figure S5a), albeit that mature Ctsb protein levels increase with age (Figure S7; Tables S29–S33). Nevertheless, GSEA of the average effect of AD pathogenesis showed an increase in the Wnt signaling pathway, with APP as the most upregulated protein (Figure 6a, Table S26). The Wnt signaling pathway, crucial for cell differentiation and muscle regeneration in adult muscle (Girardi and Le Grand 2018), is also known to regulate APP processing and is dysregulated in the brain during AD (Kostes and Brafman 2023). Elevated APP levels within muscle tissue have been suggested to play a role in brain AD pathology in APP/PS1 mice (Wu et al. 2023). GSEA of the average effect of Ctsb treatment suggested an increase in the Erk1‐Erk2 cascade, translation at presynapse, defense response and a reduction in cellular respiration, mitochondrial translation, and transport (Figure 6b). Similarly, cellular component ontologies showed a decrease in mitochondrial terms (Figure S6d).

GSEA, performed in each pairwise group comparison, suggested that many enriched ontologies were prominent in Ctsb‐treated WT mice (Figure 6c). Increased defense response, coagulation, and Erk1‐Erk2 cascade, as well as reduced mitochondrial organization, transport, and matrix, were observed in WT‐Ctsb mice. In contrast, increased translation was unique to AD‐Ctsb mice. Similar observations were found in cellular components (Figure S6e). Notably, immunoblotting of muscle Ctsb levels at shorter intervals after vector injection showed differential changes as a result of treatment in WT and AD (Figure S8; Tables S34–S43). Overall, these findings underscore the significant interplay between muscle‐derived proteins and brain pathology, suggesting that alteration in muscle proteome may play a crucial role in the progression of Alzheimer's disease.

### 3.8 Metabolic Processes in AD Plasma Are Increased by Ctsb Treatment

To elucidate the systemic effects of Ctsb treatment in the context of Alzheimer's disease, we conducted a comprehensive plasma proteome analysis. Our workflow (Figure 1b) led to the identification of 1132 proteins, of which 713 were quantified in more than three samples per group, indicating that our protocol resulted in a good coverage (Figure S6f). The classical high‐abundant plasma proteins (e.g., albumin, serpinas, apolipoproteins) were highlighted in the dynamic range curve, which covered four orders of magnitude (Figure S6g). Sample distributions did not show genotype‐ or treatment‐specific clustering (Figure S6h).

Sodium/potassium‐transporting ATPase subunit gamma (Fxyd2) was the only altered protein (FDR < 0.10) by differential abundance analysis exploring the average effect of AD (Table S27). This protein is likely to be secreted from immune cells because the Fxyd2 mRNA transcript is primarily expressed by memory CD8 T‐cells and natural killer cells (https://v19.proteinatlas.org/ENSG00000137731‐FXYD2/blood). GSEA based on biological processes suggested that metabolic and catabolic processes were decreased in AD (Figure 6d, Table S28). In contrast, blood coagulation and acute inflammatory response terms were increased, which are known to be altered in AD pathogenesis (Chen and Xia 2020). Interestingly, proteins associated with mitochondrial terms were reduced in AD (Figure S6i). However, group‐based changes showed that the overall protein abundance of mitochondrion had an increased tendency upon *Ctsb* expression in the AD group (Figure S6j). GSEA of each pairwise group comparison showed that metabolic and catabolic processes were positively enriched in the AD‐Ctsb vs. AD‐Con group, whereas negatively enriched in the AD‐Con vs. WT‐Con group (Figure 6e, Table S28). Thus, the treatment brought the proteomic profile of the AD mice closer to that of the WT control group.

Lastly, differential abundance analysis of interaction between treatment and genotype was performed. Increased enrichment of metabolic processes and intermediate filament‐based processes was detected (Table S28). Mean abundance of proteins associated with these ontologies clearly showed that AD‐WT(Con) and Ctsb‐Con(WT) groups had similar overall protein abundance (Figure 6f). These results highlight that Ctsb treatment not only influences muscle and hippocampal proteome but also enacts systemic metabolic changes that may contribute to ameliorating Alzheimer's disease symptoms.

## 4 Discussion

Increasing evidence for a role of peripheral organs in brain function has opened novel opportunities to prevent and treat neurodegenerative diseases. We hypothesized that long‐term expression of *Ctsb* in muscle, starting from around the onset of disease, may ameliorate AD‐related pathologies in APP/PS1 mice. Here we show that this treatment substantially improves memory, motor behavior, and adult hippocampal neurogenesis in AD, but not in WT mice. Proteome analysis of hippocampus, muscle, and plasma revealed specific remodeling associated with AD and Ctsb treatment. These changes may contribute to increased adult neurogenesis and thereby improve memory function in Ctsb treated AD mice.

Middle‐aged APP/PS1 mice display deficits in learning and memory function (Webster et al. 2014). In our study, AD mice acquired the water maze task slower than WT mice; however, retention of spatial memory was substantially improved by treatment, comparable to exercise interventions in middle‐aged mice (Marlatt et al. 2012). On the other hand, WT mice treated with Ctsb performed at chance levels in the probe trials, suggesting a detrimental effect of treatment on retention of spatial memory. Similarly, in the fear memory task, this group did not condition and displayed reduced cued fear, suggesting disruption of the relevant hippocampal, amygdala, and prefrontal cortex circuitry (Zelikowsky et al. 2014). While reduced freezing behavior could be attributed to increased general activity, as observed in the activity box, the impaired retention of spatial memory indicates that poor fear conditioning performance is likely due to cognitive deficits. The other groups displayed tone‐shock conditioning and cued fear, as observed in aging (Kaczorowski and Disterhoft 2009) and AD mouse (Corcoran et al. 2002) studies. Contextual conditioned fear reaction improved in treated AD mice, as it was comparable to wildtype controls. In the AD control group, contextual freezing may reflect over‐generalization (Bonardi et al. 2011). Overall, Ctsb treatment in AD mice resulted in spatial and fear memory behavior largely similar to that of the wildtype control group.

Improved memory function with Ctsb treatment in AD mice may be due in part to increased adult hippocampal neurogenesis, a highly regulatable process relevant to cognition and mood. Adult neurogenesis declines in normal aging and AD (Choi and Tanzi 2023). In our study, reduced neurogenesis in AD mice was prevented by Ctsb treatment, possibly by modulation of gene expression and protein activity within the neurogenic niche (Niklison‐Chirou et al. 2020). In particular, ribosomal dysfunction is an early event in AD pathogenesis (Ding et al. 2005). Notably, Ctsb treatment increased RNA processing and cytosolic ribosomes, suggesting restoration of the translational machinery, which contributes to improved neuronal function. Among the proteins with increased trends were P11 (S100A10), a protein that regulates the neurogenic effects of Ctsb (Moon et al. 2016), and its binding partner Anxa2. In addition, signal transducer and activator of transcription 3 (STAT3), an epigenetic regulator of neurogenic processes (Kunoh et al. 2024), and Nono, a protein important for cell cycle and synaptic function (Mircsof et al. 2015) had a similar increased tendency. Sfpq, a multifunctional RNA binding protein involved in transcriptional elongation, mRNA processing, and DNA repair, that has been reported to be downregulated in human AD brains (Younas et al. 2020), was elevated in AD‐Ctsb mice, albeit not significantly. Lastly, several proteins involved in neurogenesis and neural plasticity were regulated similarly in wildtype controls and Ctsb treated AD mice. In particular, Slc17a6, VGLUT2, displayed decreased levels following Ctsb treatment in AD mice. In line with this, we detected a trend toward reduced glutamatergic and increased GABAergic protein intensities in the hippocampus. Taken together, these data support the notion that Ctsb modulates excitotoxicity and may contribute to normalizing excitatory‐inhibitory balance (Muller et al. 2021) in the hippocampus, thereby potentially supporting improved memory function.

Differences between AD and WT in muscle proteome in response to Ctsb treatment may also contribute to behavioral and neurogenic outcomes. In WT muscle, Ctsb caused a decrease in mitochondrial and increased coagulation processes, which may be linked to deficient cognition (Oudbier et al. 2022). Our analysis shows increased cytoplasmic translation and cytosolic ribosome with Ctsb treatment in AD mice. Improved rotarod performance may be linked in part to these changes, as these processes are important for muscle growth and maintenance (Figueiredo and McCarthy 2019). Furthermore, in plasma, mitochondrial abundance and metabolic processes had an increased tendency in AD, suggesting that Ctsb has a disease‐modulating role.

### 4.1 Limitations and Future Directions

The current approaches to AD treatment are focused on diminishing neuroinflammation and reducing amyloid plaques (DeTure and Dickson 2019). While these are important targets, our study shows that even in the absence of changes in plaque pathology and neuroinflammation, muscle Ctsb treatment promotes adult hippocampal neurogenesis and memory function. Moreover, our proteomics analyses across tissues and plasma provide insight into the potential underlying mechanisms, further supporting the concept that there are novel and thus far sparsely explored signaling pathways that could be harnessed to treat the disease. However, our study has several limitations, and there are remaining questions that need to be addressed. Although amyloid plaque burden was not significantly altered by Ctsb, our proteomic data suggests a shift toward anti‐amyloidogenic processing of App in AD hippocampus, as evidenced by increased levels of α‐secretase and decreased levels of β‐secretase. This change may underlie some of the cognitive benefits and remains to be further investigated. Another observation is that the treatment benefits AD mice but is detrimental in WT. It remains to be determined why; however, proteomic analyses in WT shows increased coagulation and decreased mitochondrial function in muscle, and reduced cell chemotaxis and increased cytoskeleton pathways in hippocampus, which together may reduce neural plasticity. In addition, at shorter intervals after virus injection, there are differential changes in Ctsb processing (Ni et al. 2022; Porter et al. 2013) between WT and AD muscle. In WT, there is a reduction in the mature single‐chain protein, whereas in AD mice there is a reduction in pro‐Ctsb and an increase in the ratio between mature and immature protein. The latter is likely an indication of enhanced availability of biologically active protein in AD. We also show that with age, mature double‐chain Ctsb increases while pro‐Ctsb decreases. These findings are consistent with age‐related modifications in proteolytic proteins in mouse and *Drosophila* muscle (Hunt et al. 2021; Triolo et al. 2022). It remains to be further investigated how changes in endogenous Ctsb levels interact with vector treatment. Some limitations include the use of male mice only and reduced power due to limited sample size; however, given the promise of the results for AD intervention, further research is warranted. Furthermore, it will be important to investigate if the present findings can be extended to other AD mouse models, and whether a shorter or longer time course of administration of the vector impacts outcome. Finally, it is unknown if targeting organs other than muscle would have similar or different outcomes for the measured parameters.

Altogether, muscle Ctsb treatment in AD mice benefits adult neurogenesis and memory function, despite the presence of plaque pathology and neuroinflammation. Proteomic analyses across tissues and plasma revealed peripheral and central changes that may underlie the disease‐modifying outcomes. Muscle Ctsb has potential as a therapeutic approach for neurodegeneration.

## Author Contributions

A.P., H.H., C.M.L., A.E., C.A., O.C., A.R.W., G.M., K.J.S., T.K., A.V.K., and N.P.V. performed experiments. A.P., C.M.L., H.H., A.V.K., N.P.V., and A.A. performed data analysis. Methodology: S.G. and T.K. Conceptualization: H.V.P. Writing – original draft: A.P., H.H., C.M.L., A.S.D., and H.V.P. Writing – review and editing: A.A., A.V.K., N.P.V., R.B., and T.K. Supervision: A.S.D., R.B., and H.V.P.

## Conflicts of Interest

The authors declare no conflicts of interest.

## Supporting information


**Data S1:** acel70242‐sup‐0001‐DataS1.pdf.

**Data S2:** acel70242‐sup‐0002‐DataS2.pdf.

**Table S23:** Data matrix of the hippocampal proteome.

**Table S24:** Gene set enrichment analysis of hippocampal proteins.

**Table S25:** Data matrix of the muscle proteome.

**Table S26:** Gene set enrichment analysis of muscle proteins.

**Table S27:** Data matrix of the plasma proteome.

**Table S28:** Gene set enrichment analysis of plasma proteins.

## Data Availability

The mass spectrometry proteomics data have been deposited in the ProteomeXchange Consortium (http://proteomecentral.proteomexchange.org) via the PRIDE partner repository with the dataset identifier PXD057069.
