# Supplementary material for: Muscle Cathepsin B Treatment Improves Behavioral and Neurogenic Deficits in a Mouse Model of Alzheimer's Disease
Source: Aging Cell. 2025 Oct 5;24(11):e70242. doi: 10.1111/acel.70242 (PMC12610946; doi:10.1111/acel.70242)
Supplement: Supplementary file 1 — Data S1: acel70242‐sup‐0001‐DataS1.pdf. [file ACEL-24-e70242-s008.pdf]

Supporting Information

Muscle Cathepsin B treatment improves behavioral and neurogenic deficits in a mouse model of Alzheimer's Disease

Alejandro Pinto[1*], Hazal Haytural[2*], Cássio Morais Loss[1*], Claudia Alvarez[1], Asude Ertas[1], Olivia Curtis[1], Alyssa R. Williams[1], Grayson Murphy[1], Ken Salleng[1], Sylvia Gografe[1], Nishant P. Visavadiya[2], Andy V. Khamoui[2], Ali Altıntaş[3], Tal Kafri[4], Romain Barres[3,5], Atul S. Deshmukh[3#], Henriette van Praag[1#]

[1]Stiles-Nicholson Brain Institute, Charles E. Schmidt College of Medicine, Florida Atlantic University, Jupiter, Florida, USA.
[2]Department of Exercise Science and Health Promotion, Florida Atlantic University, Boca Raton, Florida, USA
[3]Novo Nordisk Foundation Center for Basic Metabolic Research, University of Copenhagen, DK-2200, Copenhagen, Denmark
[4]Gene Therapy Center, University of North Carolina at Chapel Hill, North Carolina, USA
[5]Institut de Pharmacologie Moléculaire et Cellulaire, Université Côte d'Azur & Centre National pour la Recherche Scientifique (CNRS), 06560 Valbonne, France

[*]Alejandro Pinto, Hazal Haytural and Cassio Loss contributed equally to this work

[#]Correspondence:

Atul S. Deshmukh
atul.deshmukh@sund.ku.dk

Henriette van Praag
hvanpraag@health.fau.edu

2. MATERIALS AND METHODS

2.1 Mice and Housing Environment

B6.Cg-Tg(APPswe,PSEN1dE9)85Dbo/Mmjax (APP/PS1) double transgenic hemizygous male mice on the C57BL/6;C3H background, and female mice without the APP/PS1 allele (referred to here as wild-type; WT) were purchased from Jackson Laboratories (MMRRC Strain #034829-JAX). Mice were group housed in Individually Ventilated Cages (Tecniplast, Emerald line) containing bedding and nesting material (nestlets), and acclimated to the vivarium environment prior to breeding. Cages were maintained in a temperature-controlled environment (21 ± 2°C) and under a 12h/12h light-dark cycle (lights were switched off at 7:30 PM), with water and food available *ad-libitum*.

All animal-use procedures were conducted after approval by Florida Atlantic University's Institutional Animal Care and Use Committee and they were conducted in accordance with the National Institutes of Health Guidelines for the care and use of Laboratory Animals.

2.2 Experimental Procedures

Breeding strategy and allocation to the groups

WT female and APP/PS1 hemizygous male mice (6-8 months old) were used as breeding pairs to produce APP/PS1 hemizygous mice (which were utilized as the AD model) and their WT littermates (serving as genotype controls). A 1:1 female-to-male ratio was employed as an in-house breeding strategy. Litters were with their respective dams until weaning at postnatal day 20-21. The weaning procedure involved ear-tagging the pups, collecting tissue (tip of the tail) for genotyping (Transnetyx), and group housing the mice (3-4 mice per cage) according to sex. No more than two mice per genotype/litter were assigned to the same treatment group. Four-month-old male mice, derived from 2 cohorts of mice, were allocated to one of the following groups for

either Ctsb or Control treatment: WT-Con (N=10 (6+4)), WT-Ctsb (N=9 (6+3)), AD-Con (N=8 (5+3)), and AD-Ctsb (N=14 (10+4)).

Outline of experiments

At four months of age, AD mice and their WT littermates, were allocated to either Ctsb or Control treatment. Ctsb was over-expressed in muscle tissue by injecting an AAV vector to express the mouse *Ctsb* gene driven by the muscle creatine kinase promoter (MCK). One month thereafter, mice were injected with bromodeoxyuridine (BrdU) intraperitoneally (i.p.) to label dividing cells. Mice were left undisturbed until the onset of behavioral testing at 10 months of age (as detailed below). Upon conclusion of behavioral testing, mice were 11.5-months old. At 12 months of age mice were deeply anesthetized for blood and tissue collection.

AAV Vector. Four-month-old mice received tail vein injections of pAAV9-tMCK-mCTSB-IRES-eGFP or control vector pAAV9-tMCK-eGFP-WPRE (VB5037) (Vector BioSystems). For tail vein injections, mice were lightly anesthetized within an induction chamber with isoflurane (Abbott) and subsequently placed onto a tail illuminator restrainer (Braintree Scientific). Once the ventral tail vein was dilated by the heat of the illuminator, 50 μL of vector (10^{11} vg/ml) was slowly injected into the blood vessel. When mice were fully recovered from anesthesia (~10 min) they were returned to their home cage.

BrdU. To evaluate long-term survival of newborn cells in the adult dentate gyrus of the hippocampus, mice were injected with BrdU, a thymidine analog that is incorporated into the DNA of dividing cells. BrdU injections occurred one month after vector treatment. Specifically, 5-month-old mice received daily i.p. injections of BrdU (50 mg/kg) for 10 consecutive days. BrdU (Sigma, B5002-1G) was prepared fresh daily by dissolving the powder in 0.9% saline at a 10

mg/mL solution concentration, followed by 30 min incubation in a 37 °C water bath, filtration through a sterile syringe at 0.2 µm, and was protected from the light prior to injection.

2.3 Behavioral tests

Upon 10 months of age mice were subjected to a battery of behavioral tests. Mice were tested in the activity box (open field) test, rotarod, Morris water maze, and fear conditioning paradigm. All tests were performed during the light period of the light-dark cycle.

2.3.1 Activity Box (Open field)

Tests were carried out in empty plexiglass arenas (height 20.3 cm, width 27.2 cm, depth 27.3 cm) containing two 16-photo beam infrared (IR) arrays on the X and Y axes. Eight arenas were used simultaneously, each one located inside a sound-attenuated chamber equipped with two ceiling white lights (SKU ENV-221CL, Version 4.0, Med Associates, St. Albans, VT, USA). Mice were acclimated to the testing room for at least 45 min before starting the test. Procedure consisted of placing the mice in the center of the arena and allowing them to move freely for 60 min. Spontaneous locomotor activity (ambulatory distance) was recorded by Activity Monitor software (Med Associates).

2.3.2 Rotarod

Motor function was evaluated with an accelerating rotarod for mice (Med Associates, St. Albans, VT). The apparatus consisted of a rotating drum (3.2 cm diameter) separated by white plexiglass matte walls, forming five 8 cm wide stations that allows for simultaneous testing of five animals. Mice were acclimated to the testing room for at least 30 min before starting the test. Procedure consisted of placing the mice on the rotating cylinder, which was turned on at a constant low speed (4 rpm). Once all five animals were positioned on the rotating cylinder, the speed was shifted from the constant 4 rpm to an accelerating speed by adding 0.12 rpm/second, changing

from 4 to 40 rpm over five min. The latency to fall was recorded by Activity Monitor software (Med Associates). If an animal did not fall during the five min of testing, a 300s latency to fall was scored for that animal.

2.3.3 Morris water maze

Spatial learning and retrieval were evaluated using the Morris water maze paradigm(Morris, 1984). The apparatus consisted of a pool (1.83m diameter) filled with water (24-26 °C), made opaque with white nontoxic paint (Tempura, Crayola), and contained a platform (20 cm x 20cm) that was hidden 1 cm below the surface of the water. The pool was surrounded by a black curtain, 60 cm from the edge of the pool to which visual cues were attached. Indirect illumination of both the pool and cues (65 lux at the center of the maze) was achieved by positioning 4 white lights equidistantly around the pool and facing the ceiling. The pool was virtually separated into 4 quadrants, called northeast (NE), northwest (NW), southeast (SE) and southwest (SW). Mice were acclimated to the testing room daily for at least 30 min before starting the test. Mice were subjected to one acquisition session per day for 20 days. Each session consisted of four 60-s trials with a 15-s inter-trial interval, in which the hidden platform was in the NE quadrant of the pool. Mice were placed at a different starting point for each trial. If the mouse found the platform, the trial was immediately terminated. Mice were placed on the platform if they did not find it within the allocated time. In either case, mice were allowed to explore the visual cues for 15-s while on the platform. Spatial memory retention was evaluated throughout two 60-s probe trials (performed 4 h and 24 h after completion of the last acquisition session) in which the platform was removed. For both probe trials, mice started in the SW quadrant. Data was collected by an automated video tracking system (Ethovision XT 17.5).

2.3.4 Fear conditioning paradigm

Pavlovian conditioning-based approach was used to evaluate fear memory. Mice were tested within a four-chamber system utilizing a near-infrared video conditioning system (MED Associates Inc., Fairfax, VT, USA) that allows for testing of four animals simultaneously. Each chamber consisted of stainless-steel walls with one transparent plexiglass door and a floor of parallel stainless-steel rods connected to a shock generator. The chamber had an overhead white light (SKU ENV-229M) to illuminate the inside (3 to 8 lux), and a speaker which was used to deliver a tone, to serve as the conditioned stimulus – CS. Each of these chambers was located within a larger noise-attenuating chamber (height 31.75 cm x width 71.12 cm x depth 59.69 cm), including a ventilation fan delivering background noise. A near-infrared camera-tracking system (MED Associates, Georgia, VT, USA) was used to automate measurement of freezing behavior (Motion Threshold (au) = 20; Detection Method = Linear; Min Freeze Duration (f) = 18). The paradigm consisted of four phases: (i) Habituation, (ii) Conditioning, (iii) Tone-cued, and (iv) Contextual phase. Mice were acclimated to the testing room for at least 1 h before test onset. Habituation (Day 1) consisted of a single 330-s session in which mice were placed within the chamber and allowed to freely explore the environment. A 1% liquinox solution was in the tray located underneath the stainless-steel rods to serve as an odor cue and to facilitate contextual perception and discrimination. Freezing behavior was measured for the entire 330-s. Mice were immediately returned to their home cages at the end of the session. The inner surfaces of the chamber and the tray were cleaned thoroughly with 70% isopropyl alcohol. Conditioning phase (Day 2) was similar to the Habituation session with the exception that a CS, tone, paired with an Unconditioned Stimulus (US), foot shock, was presented 60 seconds after the beginning of the trial. CS consisted of a tone (5000 Hz, 90 dB tone), for 30 s, that ended with the presentation of the US, a 0.5s foot shock (0.5mA) delivered 0.5s before the end of the CS (TS1). Thereafter, the

CS-US was repeated two more times (TS2 and TS3, respectively) with a 90-s inter-stimulus interval. Freezing behavior was measured during the same 30s in which the tone was on. CFR, the response evoked by the CS-US association, was considered to be formed if freezing behavior was higher during the third than the first CS-US presentation. Tone-cued phase (Day 3) was performed to evaluate long-term evocation of Pavlovian conditioning. It consisted of a single session similar to the Conditioning phase, except that no US was delivered. In addition, to avoid contextual (visual, tactile and olfactory) influence on the CS-US association, the original environment was modified by adding a blue semitransparent plastic film on top of the plexiglass chamber, an arch-shaped white plastic panel within the chamber and a white plastic panel at the base of the chamber as well as the replacement of the 1% liquinox solution by an 1% acetic acid solution. Three Contextual sessions (Day 3 - one hour after Tone-cued phase – Day 5 and Day 7; named C1, C2 and C3, respectively) were performed to evaluate long-term evocation of contextual CFR (i.e., whether freezing behavior was higher during the tests than during Day 1). These were identical to the Habituation session.

2.4 Euthanasia and Tissue collection

Upon completion of the behavioral experiments, mice were deeply anesthetized for terminal blood and tissue collection. Approximately 300 µL of trunk blood was collected from mice into EDTA-coated tubes and directly placed on ice. Within 15 min these samples were spun at (2,000 RCF) for 15 min within a temperature controlled (4 °C) centrifuge. Supernatant was collected and 2-3 aliquots (50-75 µL) were stored at -80 °C until further use. Following transcardiac perfusion with 0.9% saline (room temperature), liver, gastrocnemius, soleus, biceps and heart tissue, as well as prefrontal cortex and hippocampus from one hemisphere of the brain were dissected, immediately flash-frozen in liquid nitrogen, placed on dry ice, and stored at -80

°C. For the present study, hippocampus, gastrocnemius and plasma were utilized for proteomic analyses. The other hemisphere was placed in ice cold 4% paraformaldehyde (PFA), post-fixed for 72 hours, and subsequentially equilibrated in 30% sucrose until sectioning.

Time-course and muscle Ctsb expression. Additional cohorts of mice were utilized to monitor Ctsb levels with aging and at shorter intervals after vector injection. To assay whether Ctsb levels change with aging, mice at two months (WT, N=6; AD, N=6), three and a half months (AD-Con, N=4), five months (AD-Con, N=3) and twelve months of age (WT-Con, N=7, AD-Con, N=6) were utilized for immunoblotting. Furthermore, to assay effect of vector treatment, gastrocnemius muscle Ctsb was analyzed eight weeks after tail vein injection in WT mice (WT-Con, N=6; WT-Ctsb, N=6) at four and a half months of age, and in AD mice (AD-Con, N=9; AD-Ctsb, N=10) six to eight weeks after injection in three and a half and five-month-old mice. Mice were euthanized and tissues collected as described above.

2.5 Histology

Brain tissue

Sequential coronal sections (40 μm) were taken using a freezing microtome (HM450, ThermoFisher Scientific, Waltham, MA, USA) throughout the rostrocaudal extent of one hemisphere of the brain and stored in 96-well plates in a phosphate-buffered glycerol anti-freezing solution at -20 °C, as described(Zhao and van Praag, 2020).

DCX and BrdU immunohistochemistry For both DCX, an endogenous marker for immature neurons(Brown et al., 2003), and BrdU staining, sections were subjected to the following: (i) quenching endogenous peroxidase activity, (ii) antigen retrieval, (iii) immunolabeling, (iv) detection and dehydration.

DCX staining

A one-in-twelve series (480 μm apart) of free-floating sections was rinsed in 0.1M Tris-Buffered Saline (TBS, 3 x 5-min). (i) sections were incubated for 30 min (at room temperature (RT)) in 0.6% H_2O_2 in TBS to quench endogenous peroxidase activity. Five-min rinses in TBS were performed until no bubbles were detected. (ii) Antigen retrieval: incubation in 1mM citrate buffer (pH 6.0) at 95-98 °C for 20 min. (iii) Sections were rinsed 3 x 5-min in TBS followed by 1h (at RT) in blocking solution (3% donkey serum and 0.1% Triton x-100 in TBS (TBS^{++})), and overnight incubation at 4 °C in TBS^{++} containing the primary antibody (mouse monoclonal anti-DCX, 1:1000, Santa Cruz Biotechnology Cat# sc-271390, RRID:AB_10610966). Next, immunolabeling steps were repeated, albeit sections were incubated for 2h at RT with the secondary antibody (Biotin-SP goat anti-mouse, 1:500, Jackson ImmunoResearch Labs Cat# 115-065-166, RRID:AB_2338569). (iv) The detection steps: TBS (rinses 3x 5 min) followed by 2h incubation (at RT) in avidin-biotin-peroxidase complex (VECTASTAIN® Elite® ABC-HRP Kit - Vector Laboratories), TBS (3 x 5-min), a 7 min incubation in diaminobenzidine (DAB) chromogenic substrate and urea hydrogen peroxide (Sigma-Aldrich # D4418), and TBS (5x 5-min) rinses. Sections were mounted onto gelatin subbed slides, dehydrated using increasing alcohol concentrations and CitriSolv (Fisher Scientific 04-355-121), and cover-slipped with DPX mounting media (Sigma-Aldrich # 06522).

BrdU staining A one-in-six series (240 μm apart) of free-floating sections were subjected to (i) quenching of endogenous peroxidase activity (as described for DCX staining). (ii) Antigen retrieval consisted of incubating the sections in 50% formamide diluted in 2x SSC buffer (30mM sodium citrate in 0.3M NaCl) at 65 °C for 2 h, followed by 5 min incubation in 2X SSC buffer at RT, 60 min in 2N HCl at 37 °C, and 10 min incubation in 0.1M borate buffer (pH 8.5) at RT. (iii) Immunolabeling and (iv) detection and dehydration steps were identical to DCX staining, except

for the primary (rat monoclonal anti-BrdU, 1:500, Abcam Cat# ab6326, RRID:AB_305426) and secondary antibodies (Biotin-SP polyclonal donkey anti-rat, 1:500, Jackson ImmunoResearch Labs Cat# 712-065-153, RRID:AB_2315779).

DCX^+ cells and $BrdU^+$ cells were quantified from the rostral to caudal dentate gyrus. Detailed information is presented in Supplementary Material (Supplementary Information S3). In order to minimize detection bias, a computer-based random order generator was used to predefine the order in which each slide was analyzed (slide IDs were coded) by an experimenter who was blinded to the groups. The average number of positive cells per section was calculated.

Thioflavin-S Staining

Thioflavin-S (ThioS) working solution (Sigma-Aldrich: T1892) was prepared at a final 0.01% concentration diluted in 50% ethanol. A one-in-twelve series of free-floating sections (40 μm) was rinsed in TBS (3 x 5-min) followed by 30 min incubation in 0.01% ThioS solution, 5 min incubation in 70% ethanol, and rinses in dH_2O (3 x 5 min). Sections were mounted on gelatin subbed slides, and coverslipped with DABCO-PVA.

Sections were systematically imaged on an epifluorescence microscope (Olympus BX51) and quantified. For each section, one image was taken of the following brain areas: the dentate gyrus (DG) (granule cell, polymorphic and molecular layer), CA1 (pyramidal cell layer, stratum oriens, radiatum, and lacunosum moleculare), auditory/somatosensory cortex (primary/secondary auditory/somatosensory cortex), perirhinal/entorhinal cortex (perirhinal and/or dorsolateral entorhinal cortex) and cingulate cortex (retrosplenial dysgranular cortex and retrosplenial granular cortex). The "interactive learning and segmentation toolkit" (Berg et al., 2019) was used to conduct unbiased image segmentation. Briefly, after extensive training of Ilastik to identify pixels as either positive- or negative-ThioS labeling (background), a pixel-based classification was applied to all

images. A ThioS$^+$ plaque was defined as any green fluorescent labeling above the background (using an image derived from WT tissue as ThioS-negative comparison – background control). Binary output images of ThioS$^+$ labeling were exported through the "simple segmentation" feature. The "Measure" feature on ImageJ software (Image J2: Version 2.14.0/1.54f) was used for collecting data from the simple segmentation images. The estimation of the density of ThioS$^+$ plaques (% area covered/0.32 mm^2) was calculated either for each individual region and combined for hippocampus and cortex. ThioS$^+$ plaques were also counted for each 0.32 mm^2 area using the multi-point tool on ImageJ software (version 1.54g). A ThioS$^+$ plaque was defined as any green fluorescent labeling above the background (using an image derived from WT tissue as ThioS-negative comparison – background control). For the cases in which two or more green fluorescent areas were connected to each other (forming a single "ramified" area), this was considered a single ThioS$^+$ plaque. Finally, the estimation of the number of ThioS$^+$ plaques/mm^2 was calculated for each individual region and pooled for hippocampus and cortex. A computer-based random order generator used to predefine the order in which each slide was imaged and analyzed (slide IDs were coded) by experimenters who were blinded to the groups during the whole procedure.

GFAP and Iba1 immunofluorescence

A one-in-twelve series of free-floating sections (40 μm) was rinsed in TBS (3 x 5-min) and incubated for 1h in TBS^{++} (at RT). Thereafter, sections were incubated in TBS^{++} containing a cocktail of primary antibodies for 72 h at 4 °C (rabbit polyclonal anti-GFAP, 1:1000, Dako Cat# Z0334, RRID:AB_10013382; goat polyclonal anti-Iba1, 1:500, Abcam Cat# ab5076, RRID:AB_2224402). After rinses with TBS (3x) the sections were incubated (at RT) in TBS^{++} containing their respective secondary antibodies for 2 h (Alexa Fluor 488 donkey anti-rabbit, 1:500, Jackson ImmunoResearch Cat# 711-545-152, RRID: AB_2313584; Cy3 donkey anti-goat,

1:500, Jackson ImmunoResearch Cat# 705-165-147, RRID: AB_2307351). Sections were rinsed with TBS (3 x 5 min), incubated with DAPI solution (0.5 µg/ml; Molecular Probes: MP01306) for 5 min, rinsed (3 x 5 min), mounted onto gelatin-coated slides and cover-slipped with DABCO-PVA.

To quantify astrocytes ($GFAP^+$) and microglia ($Iba1^+$) area, tile scan images covering the entire hippocampal formation were acquired on a confocal microscope (Nikon, A1R). Similar to the procedure described in ThioS analysis, Ilastik (version 1.4.0.post1-gpu)(Berg et al., 2019) was used to conduct unbiased image segmentation on "max projection" confocal output images. Briefly, after extensive training of Ilastik to identify pixels as either positive or negative (background) immunofluorescent labeling, a pixel-based classification was applied to all images. To avoid miss-estimation of either $GFAP^+$ or $Iba1^+$ immunolabeling due to superimposition of pixels, independent training was conducted for each channel. Binary output images of either $GFAP^+$ or $Iba1^+$ immunolabeling were exported through the "simple segmentation" feature. The density of $GFAP^+$ and $Iba1^+$ labeling in the hippocampus was estimated by systematically sampling two 0.15 mm^2 Region of Interests (ROI) per binary image, one in the DG (containing the polymorph layer, the granule cell layer and the molecular layer) and one in the CA1 subfield (containing the pyramidal cell layer, the stratum oriens, the stratum radiatum, and the stratum lacunosum moleculare). Unbiased ROI positioning was conducted using the DAPI channel as reference in ImageJ software (Image J2: Version 2.14.0/1.54f) and applied to their respective simple segmentation images for data collection utilizing the "Measure" feature. Finally, estimation of the density of $GFAP^+$ and $Iba1^+$ labeling (% area covered/0.15 mm^2) was calculated either for each individual region or for all of them pooled together. Both performance and detection biases

were minimized by using this approach combined with experimenters who were blinded to the groups to conduct the whole procedure.

2.6 Western blotting

Gastrocnemius muscle lysate preparation

The protein lysate from the gastrocnemius muscle was prepared in Pierce™ RIPA buffer (cat# 89900, Thermo Fisher Scientific) containing protease and phosphatase inhibitor cocktails (cat# 5872, Cell Signaling Technology). The protein content of the lysate was quantified using the Pierce™ BCA Protein Assay Kit (cat# 23225, Thermo Fisher Scientific) and then stored at −80 °C until analysis. The sample lysate was prepared for Western blotting by adding 50 μg of protein lysate to Laemmli sample buffer (cat# 1610747, Bio-Rad Inc.) containing 2-mercaptoethanol as a reducing agent, followed by separation with sodium dodecyl sulfate-polyacrylamide gel electrophoresis (SDS-PAGE), using Any kD™ Criterion™ TGX Stain-Free™ Protein Gel (cat# 5678124, Bio-Rad Inc.). The gel was run at 140 V for 100 min in Tris/Glycine/SDS buffer, pH 8.3 (cat# 1610732, Bio-Rad Inc.). Subsequently, the separated and resolved proteins were electrotransferred onto a Trans-Blot Turbo Midi 0.2 μm Nitrocellulose Transfer membrane (cat#1704159, Bio-Rad Inc.) using a standard protocol (at current: 1.0 A; 25 V constant for 30 min) of the Trans-Blot® turbo™ transfer system (Bio-Rad Inc.), according to the manufacturer's instructions. After the proteins were transferred, the nitrocellulose membrane was blocked with 5% non-fat dry milk in TBST buffer, pH 7.4, containing 0.1% Tween-20 (Tris-buffered saline, cat# 1706435, Bio-Rad Inc.) for 1 h at room temperature. The membrane was then incubated overnight with anti-Cathepsin B primary antibody [CA10] (1:1000 dilution, cat# ab58802, Abcam Inc.) at 4 °C in 5% non-fat milk. This antibody allows for the detection of pro- and mature (single-chain (SC) and double-chain (DC)) isoforms(Porter et al., 2013). A rabbit monoclonal antibody

against GAPDH (1:5000 dilution, cat# 5174, Cell Signaling Inc.) was used as the internal loading control. After overnight incubation, the membrane was washed 3X with TBST, then incubated with horseradish peroxidase (HRP)-conjugated secondary antibody for 2 h at room temperature. Subsequently, the membrane was washed 3X in TBST, incubated for 5 min in Pierce™ SuperSignal™ West Pico PLUS Chemiluminescent Substrate (cat# 34580, Thermo Fisher) for protein detection, and the reactive bands were then identified and visualized using ChemiDoc^{TM} XRS+ imager system with Image Lab™ software (Bio-Rad Inc.). The band densities of pro-, and mature (SC, DC) forms were determined using NIH ImageJ software.

Hippocampal lysate preparation

Hippocampal tissues were lysed in 4% Sodium Dodecyl Sulfate (SDS) buffer (100 mM Tris, pH 8.5) using BeatBox Tissue Homogenizer (PreOmics). Lysates were then boiled at 95 ^{0}C for 10 min, followed by 10 min sonication in ultrasonic bath sonicator. After centrifugation at 16000 g for 10 min, supernatants were transferred into a new tube for determination of protein concentration using DC protein assay (Thermo Fisher Scientific). Sample buffer (containing 2-mercaptoethanol) was added to the hippocampal lysates using 1:4 ratio, and 9 µg of lysates were loaded onto the 4-20% Criterion^{TM} TGX^{TM} Precat midi protein gel (Bio-Rad). The gel was run at 100 V for 15 min, followed by 150 V for 45 min in 1X Tris/Glycine/SDS buffer (Bio-Rad Inc.). Proteins were transferred to Trans-Blot Turbo Midi 0.2 µm PVDF membrane (Bio-Rad Inc.) using a standard protocol for mixed molecular weight (25 V for 7 min) at the Trans-Blot® turbo™ transfer system (Bio-Rad Inc.). The membrane was blocked with 5% non-fat dry milk in TBS-T buffer, pH 7.4, containing 0.1% Tween-20 for 45 min at room temperature. The membrane was then incubated overnight at 4 °C with primary antibodies prepared in 2% milk: anti-vGLUT2 antibody (guinea pig, 1:200 dilution, ab_2571621, Frontier Institute), anti-ANXA2 (rabbit, 1:1000 dilution, 8235,

Cell Signaling Technology), or anti-GAPDH (rabbit, 1:5000 dilution, Cell Signaling Technology). GADPH was used to assess protein loading. Next day, the membrane was washed 3X with TBS-T, then incubated with horseradish peroxidase (HRP)-conjugated secondary antibodies for 1 h at room temperature. Subsequently, the membrane was washed 3X in TBS-T, incubated for 1 min in Immobilon Forte Western HRP substrate (Merck) and imaged using ChemiDoc™ XRS+ imager system with Image Lab™ software (Bio-Rad Inc.). The adjusted volume densities for each band were detected by Image Lab™ software.

2.7 Proteomics

Sample preparation for proteomics analysis

Hippocampal and gastrocnemius muscle tissues were powdered and lysed in 4% Sodium Dodecyl Sulfate (SDS) buffer (100 mM Tris, pH 8.5) using Ultra Turrax blender (IKA). Lysates were then boiled at 95 ^{0}C for 10 min, followed by 10 min sonication in ultrasonic bath sonicator. After centrifugation at 16000 g for 10 min, supernatants were transferred into a new tube for determination of protein concentration using DC protein assay (Thermo Fisher Scientific). Subsequently, 20 µg of proteins were reduced by the addition of 100 mM of dithiothreitol and alkylated by 40 mM of chloroacetamide. After 45 min of incubation at the room temperature, proteins were digested using PAC protocol on KingFischer Flex robot (Batth et al., 2019). In brief, a 1:4 protein to bead ratio was added to the sample lysate and protein aggregation was induced by dispensing a final concentration of 70% acetonitrile (ACN), followed by a 10-min incubation without agitation. Then, the sample plates were placed in a magnetic stand (Dynamag Thermo) and beads with protein aggregates were first washed twice with 100% ACN and then twice with 70% ethanol. After discarding the last wash, beads were resuspended in 100 µl of digestion buffer (50 mM Tris-HCl, pH 8.5) containing 1:100 enzyme to protein ratio of trypsin and 1:500 enzyme to protein ratio

of LysC (Wako) and enzymatic digestion was done at 37 °C overnight. The next day, digestion was quenched by addition of a final concentration of 1% trifluoroacetic acid (TFA) in isopropanol. Peptides were then cleaned for salts and remaining lipids using styrenedivinylbenzene–reverse phase sulfonate (SDB-RPS, details) stage-tips and eluted in 60 µl of 1% ammonia and 80% ACN. Using speedvac, peptides were dried completely and resuspended in 10 µl of 0.1% TFA and 5% ACN. Peptide concentration was determined by a NanoDrop spectrophotometer (Thermo Fisher Scientific) and a total of 200 ng of peptides were loaded on Evotip C18 trap columns (Evosep Biosystems) according to the manufacturer's instructions.

For the plasma proteomics, 1 µl of plasma was directly lysed in 100 µl of 100 mM Tris buffer (pH 8.5). Lysates were then boiled at 95 ^{0}C for 10 min, followed by 10 min sonication in ultrasonic bath sonicator. After a quick spin down, enzyme mix (containing 1:100 enzyme to protein ratio of trypsin and 1:500 enzyme to protein ratio of LysC) were added and enzymatic digestion was done at 37 °C overnight. The next day, digestion was quenched by addition of a final concentration of 2% TFA. Peptide concentration was then determined by a NanoDrop spectrophotometer (Thermo Fisher Scientific) and a total of 350 ng of peptides were loaded on Evotip C18 trap columns (Evosep Biosystems) according to the manufacturer's instructions.

LC-MS/MS analysis

Peptides of hippocampus and gastrocnemius muscle samples were separated on 15-cm, 150-µM ID column packed with C18 beads (1.5 µm) (Pepsep) on an Evosep ONE HPLC system applying the default 30-SPD (30 samples per day) method. Column temperature was held at 50 ^{0}C. Upon elution, peptides were injected via a CaptiveSpray source and 20-µm emitter into a timsTOF Pro 2 mass spectrometer (Bruker) operated in diaPASEF mode. MS data were collected over a 100-1700 m/z range. During MS/MS data collection each diaPASEF cycle was 1.8 seconds, covering the ion

mobility range of 1.6-0.6 1/K0. Ion mobility was calibrated using three Agilent ESI-L Tuning Mix ions 622.0289, 922.0097 and 1221.9906. For diaPASEF we used a long-gradient method which included 10 diaPASEF scans with three 25 Da windows per ramp (for hippocampus) whereas 16 diaPASEF scans with two 25 Da windows per ramp (for muscle), mass range 400.0-1201.0 Da and mobility range 1.43-0.60 $1/K_0$. The collision energy was decreased linearly from 59 eV at $1/K_0 = 1.3$ to 20 eV at $1/K_0 = 0.85$ Vs cm^{-2}. Both accumulation time and PASEF ramp time was set to 100 ms. For the plasma proteomics, peptides were separated on a PepSep 8 cm, 150 µM ID reversed-phase column packed with C18 beads (1.5 µm) (PepSep) using an Evosep One LC system with the default 60-SDP (60 samples per day) method. Column temperature was maintained at 35 °C. Liquid chromatography was coupled via a CaptiveSpray ion source with a timsTOF Pro2 (Bruker Daltonics) operating in diaPASEF mode. MS data were collected over a 100-1700 m/z range. During each MS/MS data collection each dia-PASEF cycle was 0.95 seconds, covering the ion mobility range 1.4-0.6 $1/K_0$. Ion mobility was calibrated using three Agilent ESI-L Tuning Mix ions 622.0289, 922.0097, and 1221.9906. For diaPASEF we used a modified version of the short-gradient method which included 8 diaPASEF scans with three 25-Da windows per ramp, mass range 400.0-1000.0 Da, and mobility range 1.37-0.64 $1/K_0$. The collision energy was decreased linearly from 45 eV at $1/K_0 = 1.3$ to 27 eV at $1/K_0 = 0.85$ Vs cm-2. Both accumulation time and PASEF ramp time were set to 100 ms.

MS data analysis

Raw MS data was quantified using DIA-NN software v.1.8.1 (Demichev et al., 2020). The precursor mass and fragment mass were matched with an initial mass tolerance of 10 and 20 ppm, respectively. The search also included the following default parameters. The fixed modification of carbamidomethyl cysteine and N-terminal methionine excision were enabled. Peptide length was set

to seven to 30 amino acids, and missed miscleavages were set to one. The precursor m/z range was set from 300 to 1800. The false discovery rate (FDR) was set to 1% at the peptide precursor level and protein level, and match between runs was enabled.

Differential proteome analysis

Proteomic data analysis was performed in R (v.4.3.0) with the following packages: clusterProfiler (v4.8.3), dplyr (v1.1.4), ggplot2 (v3.5.1), factoextra (v 1.0.7), limma (v3.56.2), PhosR (v1.10.0), plotly (v4.10.4), reshape2 (1.4.4), viridis (v0.6.5). Protein intensities were transformed to log2 scale, samples with low protein identifications (< 50 %) were removed, missing values were filtered to allow at least 60% of quantified values within each experimental group (WT-Con, WT-Ctsb, AD-Con and AD-Ctsb), and then quantile normalization was performed. In hippocampal and muscle proteomics, missing values were imputed from a normal distribution using tImpute function (width: 0.3, down shift: 1.8) by PhosR package. Subsequently, variance was corrected using weighted surrogate variance analysis (wsva) function from the limma package(Leek and Storey, 2007). Differential expression analysis was done using lmFit function by limma package. Contrasts for different pairwise group comparisons, the main effects of CTSB treatment and AD pathogenesis as well as the interaction between CTSB and AD pathogenesis were assessed. Using Benjamini-Hochberg method, p values were corrected and FDR < 0.10 was considered as a cut-off for statistical significance. Gene set enrichment analyses were done by the ClusterProfiler package using biological process, cellular component and molecular function ontologies separately. Gene sets with adjusted p values < 0.05 were considered statistically significant. Gene ontology terms called synaptic transmission, glutamatergic (GO: 0035249) and synaptic transmission, GABAergic (GO: 0051932) were downloaded from Quick GO (https://www.ebi.ac.uk/QuickGO/).

2.8 Statistical analysis

Statistical analyses were performed as described(Loss et al., 2021). All behavioral and histological data was analyzed in RStudio (Version 1.4.1717 – 2009-2021 RStudio, PBC) R version 4.1.0 (2021-05-18) using either Generalized Linear Model (GLM) or Generalized Linear Mixed Model (GLMM). The family distribution and its canonical link function were chosen beforehand taking in account the nature of the data(Schmettow, 2021). Specifically, continuous data with no boundaries were analyzed using Gaussian distribution with Identity link; continuous data presenting no upper boundaries but presenting lower boundaries = zero were analyzed using Gamma distribution with Log link; continuous data presenting both upper and lower boundaries were analyzed using Beta distribution with Logit link; discrete data (counts) presenting only lower boundaries = zero were first analyzed using Poisson distribution with Log link followed by Negative Binomial distribution with Log link for the cases in which over-dispersion was detected during diagnosis procedure. GLM and GLMM were performed using either lme4 package(Bates, 2015) or glmmTMB package (Brooks, 2017). When necessary, the raw data were subjected to transformations before the analysis, as described for each analysis in the Supplementary Material.

A backward stepwise selection model approach (based on the Akaike Information Criterion – AIC – through the use of the stepAIC function) or, alternatively, an approach evaluating all possible combinations of subsets of factors (based on the Akaike Information Criterion corrected for small samples – AICc – through the use of the dredge function from the MuMin package) was applied to select the most parsimonious model. The model presenting the lowest AIC (or AICc) value was selected. All selected models were submitted to diagnosis using the DHARMa package(Hartig, 2022). Except for overdispersion in the models using Poisson distribution (as described above), no action was made to avoid diagnosis problems (diagnosis outputs are

described for each individual variable in Supplementary Material). Estimated marginal means and 95% CI were extracted from the selected models using emmeans package(Lenth, 2022) and were graph plotted using GraphPad Prism 8. Bonferroni pairwise (post hoc) comparisons were performed for the cases in which any interaction effect was detected, controlling for family-wise error. For time spent in target quadrant data (probe trials in the MWM paradigm), an additional analysis was performed in which each group (WT-Con, WT-Ctsb, AD-Con, and AD-Ctsb) was individually compared against the 25% chance of randomly staying in each of the quadrants (One-Sample Wilcoxon Signed Rank Test). Correlations were evaluated by Spearman's rank correlation. A 0.05 significance level (alpha) was set for all analyses. All the statistical results are presented in detail in the Supplementary Tables.

2.9 Bias-reducing measures

Randomization, blinding and systematic sampling procedures were applied to minimize both performance and detection bias for all histological data. Detection bias was further minimized by using automated systems (equivalent to blinding outcome assessors) to collect all behavioral data. Attrition bias was minimized by adhering to all predefined exclusion criteria as follows: (i) identification of signs of pain or distress in the mice (no animals were excluded based on this criterion, however, one AD-Ctsb mouse died between the last behavioral testing session and euthanasia). (ii) Poor staining/image quality resulted in exclusion of 1 animal (WT-Ctsb) in DCX analysis; 2 animals (1 WT-Con and 1 AD-Ctsb) in GFAP/Iba1 analysis; 3 AD-Ctsb mice in ThioS analysis. No outliers were excluded. Based on “ARRIVE guidelines 2.0: Essential 10 list”(Percie du Sert et al., 2020) available at https://arriveguidelines.org/arrive-guidelines and on “SYRCLE’s risk of bias tool for animal studies”(Hooijmans et al., 2014) all efforts have been made to ensure transparency and avoid reporting bias(Loss et al., 2021).

References

Bates, D., Mächler, M., Bolker, B., & Walker, S. (2015). Fitting Linear Mixed-Effects Models Using lme4. . Journal of Statistical Software *67*, 1-48.

Batth, T.S., Tollenaere, M.X., Ruther, P., Gonzalez-Franquesa, A., Prabhakar, B.S., Bekker-Jensen, S., Deshmukh, A.S., and Olsen, J.V. (2019). Protein Aggregation Capture on Microparticles Enables Multipurpose Proteomics Sample Preparation. Mol Cell Proteomics *18*, 1027-1035.

Berg, S., Kutra, D., Kroeger, T., Straehle, C.N., Kausler, B.X., Haubold, C., Schiegg, M., Ales, J., Beier, T., Rudy, M., *et al.* (2019). ilastik: interactive machine learning for (bio)image analysis. Nat Methods *16*, 1226-1232.

Brooks, M.E., Kristensen, K., Van Benthem, K. J., Magnusson, A., Berg, C. W., Nielsen, A., et al. (2017). glmmTMB balances speed and flexibility among packages for zero-inflated generalized linear mixed modeling. The R Journal *9*, 378-400.

Brown, J.P., Couillard-Despres, S., Cooper-Kuhn, C.M., Winkler, J., Aigner, L., and Kuhn, H.G. (2003). Transient expression of doublecortin during adult neurogenesis. J Comp Neurol *467*, 1-10.

Demichev, V., Messner, C.B., Vernardis, S.I., Lilley, K.S., and Ralser, M. (2020). DIA-NN: neural networks and interference correction enable deep proteome coverage in high throughput. Nat Methods *17*, 41-44.

Hartig, F. (2022). DHARMa: Residual Diagnostics for Hierarchical (Multi-Level /Mixed) Regression Models. R package version 0.4.5. .

Hooijmans, C.R., Rovers, M.M., de Vries, R.B., Leenaars, M., Ritskes-Hoitinga, M., and Langendam, M.W. (2014). SYRCLE's risk of bias tool for animal studies. BMC Med Res Methodol *14*, 43.

Leek, J.T., and Storey, J.D. (2007). Capturing heterogeneity in gene expression studies by surrogate variable analysis. PLoS Genet *3*, 1724-1735.

Lenth, R.V. (2022). emmeans: Estimated Marginal Means, aka Least-Squares Means. R package version 1.7.3. .

Loss, C.M., Melleu, F.F., Domingues, K., Lino-de-Oliveira, C., and Viola, G.G. (2021). Combining Animal Welfare With Experimental Rigor to Improve Reproducibility in Behavioral Neuroscience. Front Behav Neurosci *15*, 763428.

Morris, R. (1984). Developments of a water-maze procedure for studying spatial learning in the rat. J Neurosci Methods *11*, 47-60.

Percie du Sert, N., Hurst, V., Ahluwalia, A., Alam, S., Avey, M.T., Baker, M., Browne, W.J., Clark, A., Cuthill, I.C., Dirnagl, U., *et al.* (2020). The ARRIVE guidelines 2.0: updated guidelines for reporting animal research. J Physiol *598*, 3793-3801.

Porter, K., Lin, Y., and Liton, P.B. (2013). Cathepsin B is up-regulated and mediates extracellular matrix degradation in trabecular meshwork cells following phagocytic challenge. PLoS One *8*, e68668.

Schmettow, M. (2021). Generalized Linear Models. In New Statistics for Design Researchers A Bayesian Workflow in Tidy R (Germany: Springer International Publishing), pp. 323-399.

Zhao, X., and van Praag, H. (2020). Steps towards standardized quantification of adult neurogenesis. Nat Commun *11*, 4275.
