# Supplementary material for: Muscle Cathepsin B Treatment Improves Behavioral and Neurogenic Deficits in a Mouse Model of Alzheimer's Disease
Source: Aging Cell. 2025 Oct 5;24(11):e70242. doi: 10.1111/acel.70242 (PMC12610946; doi:10.1111/acel.70242)
Supplement: Supplementary file 2 — Data S2: acel70242‐sup‐0002‐DataS2.pdf. [file ACEL-24-e70242-s005.pdf]

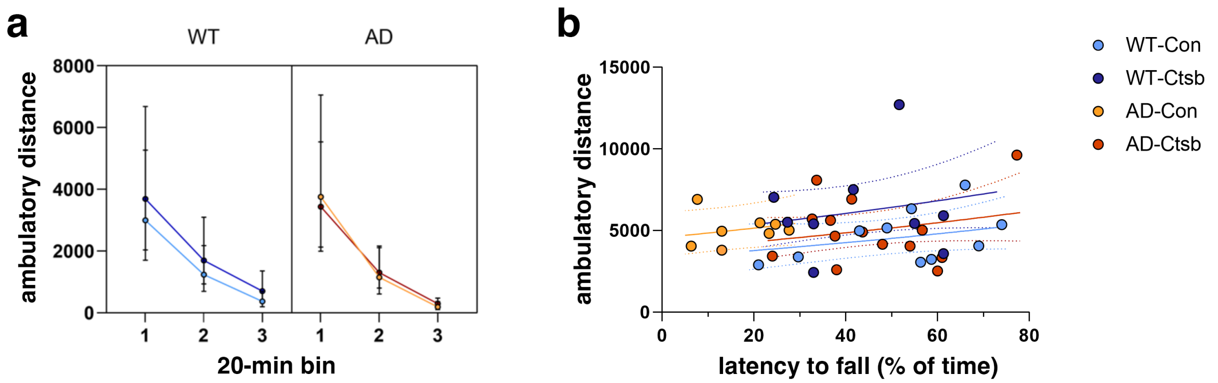

Fig. S1. Activity Box. (a) Ctsb treatment increased the distance traveled in the activity box (open field) in WT-Ctsb mice (Table S1). Analysis of the ambulatory distance over time (20 min bins) revealed a Time effect (GLMM analysis) in which Bin 1 > Bin 2 > Bin 3 for all groups (Table S2). This suggests that all groups habituated similarly to the arena and that between group differences in total ambulatory distance are a result of distinct activity levels. **(b)** Generalized Linear Model (GLM) analysis revealed an interaction between genotype and treatment, and a latency to fall effect that indicates a within-group relationship in which mice that fall sooner in the rotarod traveled a shorter distance than mice that fall later (Table S1). Solid lines represent the Estimated Marginal Means while dashed lines represent 95% CI. (N; WT-Con = 10, WT-Ctsb = 9, AD-Con = 8, AD-Ctsb = 14). Data are presented as Estimated Marginal Means and their respective 95% CI.

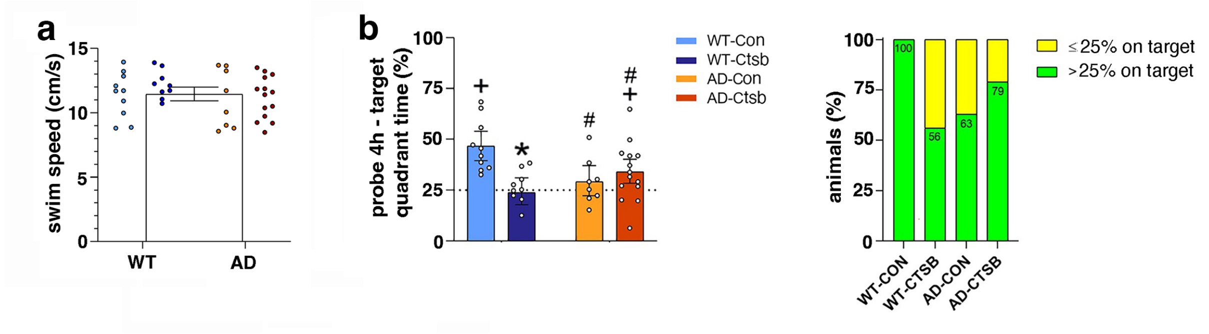

Fig. S2. Morris water maze performance. (a) Swim speed. Average swim speed did not differ between the groups on acquisition day 1 (Table S5) and did not change over time (Table S6). The single bar and whiskers represent the Estimated Marginal Means and its 95% CI (respectively) estimated by the model for the entire data set (i.e., all groups pooled together). **(b)** Target quadrant time in the 4 h probe trial. In the probe trial the WT-Con group spent more time in the target quadrant than the WT-Ctsb and AD-Con groups. AD-Ctsb mice spent more time in the target quadrant than WT-Ctsb mice. Indeed, both the WT-Con and AD-Ctsb groups preferred the target quadrant above 25% chance, indicating that these groups remember the location of the platform that was present during the acquisition phase. Specifically, WT-Con and AD-Ctsb mice were in the target quadrant 46% ($p<0.001$) and 34% ($p=0.024$), respectively, of the time, whereas the AD-Con mice and WT-Ctsb spent 29% ($p=0.19$) and 23% ($p=0.36$) of the time, respectively, and as depicted in the contingency graph of the percentage of mice that spent either more (in green) or less (in yellow) than 25% of the time in the target quadrant (Table S8). (N; WT-Con = 10, WT-Ctsb = 9, AD-Con = 8, AD-Ctsb = 14). Data were analyzed by GLM and are presented as Estimated Marginal Means and their respective 95% CI. $^{\#}$P<0.05 AD compared to WT in the same Treatment. *P<0.05 Ctsb compared to Con in the same Genotype. $^{+}$P<0.05 compared to 25% chance (one-tailed Wilcoxon signed rank exact test).

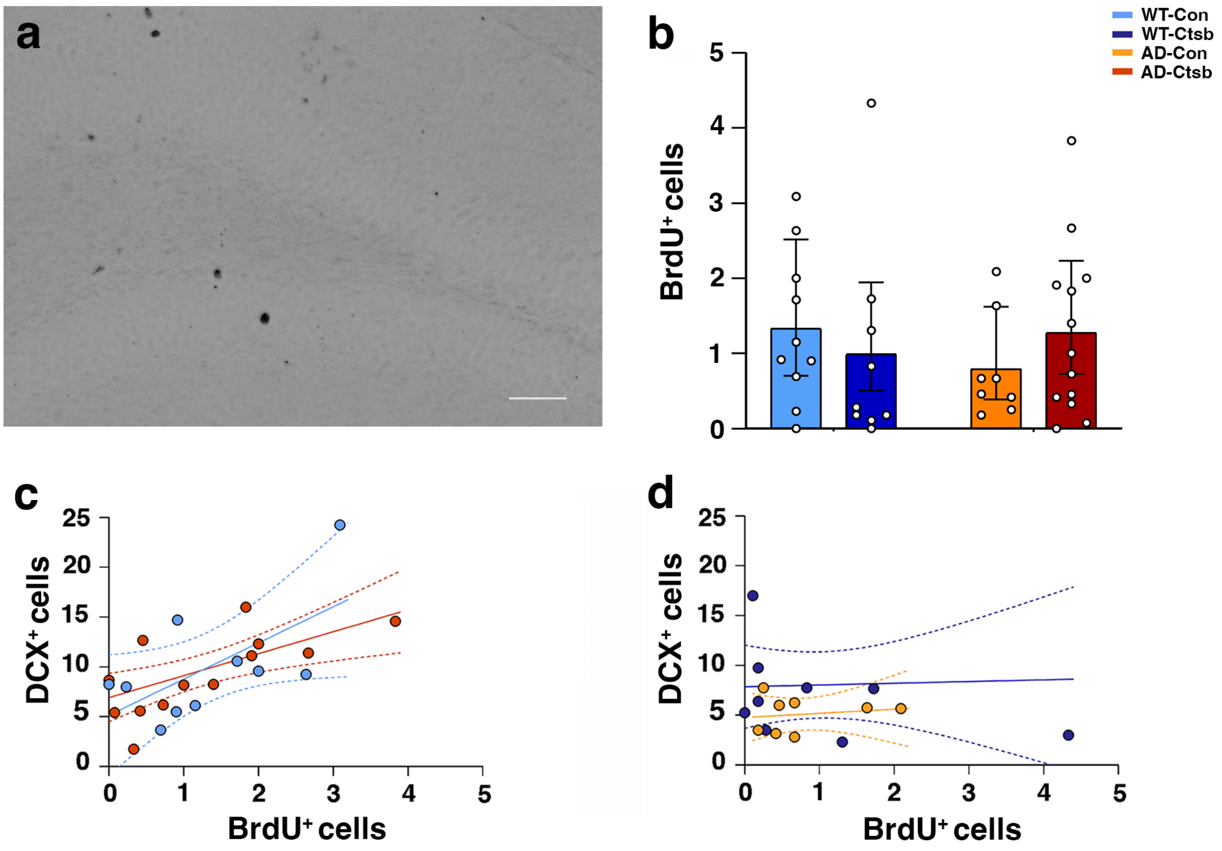

Fig. S3. Long-term adult-born cell survival and correlations between DCX and BrdU. (a) Representative photomicrograph of BrdU labeling in the dentate gyrus. Scale bar 50 µm. **(b)** BrdU$^+$ cells per section did not differ between the groups (mean = 1.13; 95% CI 0.84 – 1.54; Table S15). **(c,d)** Spearman correlation analysis between DCX$^+$ cells and BrdU$^+$ cells (Table S16). **(c)** BrdU$^+$ and DCX$^+$ cell number increased concurrently in both WT-Con (P=0.0665) and AD-Ctsb (P= 0.0183) groups, but not in (D) WT-Ctsb (P= 0.35) and AD-Con (P= 0.91) groups. In **b**, data were analyzed by GLM and are presented as Estimated Marginal Means and their respective 95% CI. In **c** and **d**, solid lines represent the correlation for each group while dashed lines represent their respective 95% CI.

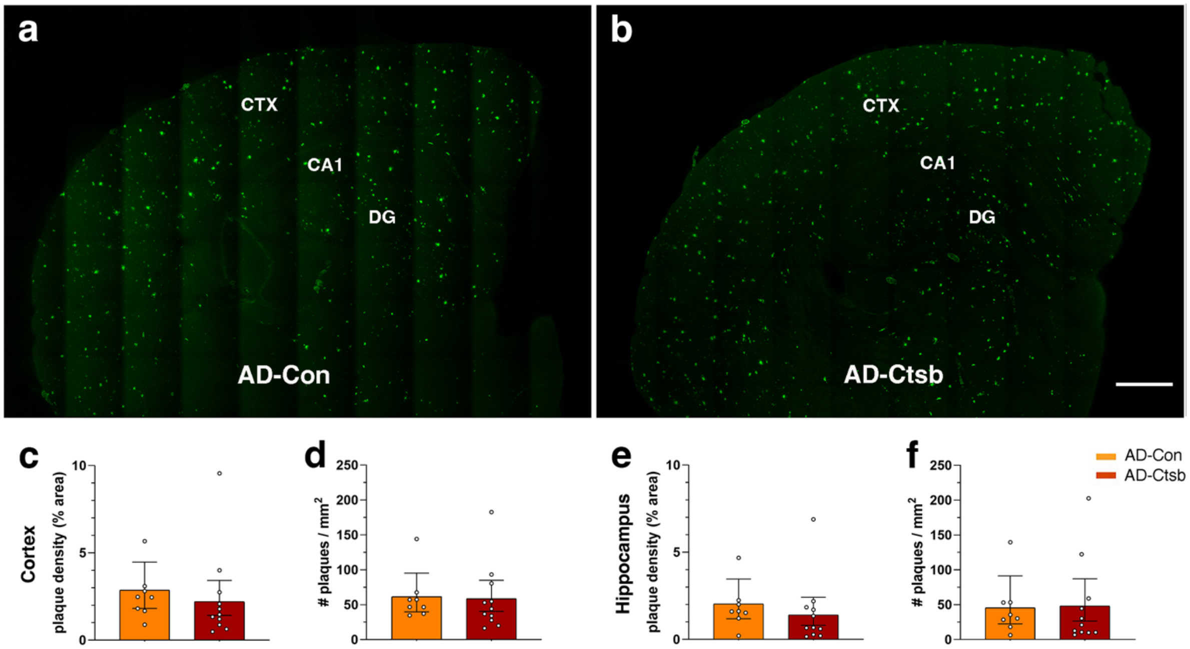

Fig S4. Ctsb treatment does not change amyloid plaque deposition. (a,b) Representative photomicrographs of ThioS staining (green) in sections derived from **(a)** AD-Con and **(b)** AD-Ctsb brain tissue. Scale bar: 500 µm. **(c-f)** There was no difference between AD-Ctsb and AD-Con mice in ThioS^{+} amyloid plaque density or number in **(c,d)** cortex or **(e,f)** hippocampus. Data were analyzed by GLM and are presented as Estimated Marginal Means and their respective 95% CI. (N; AD-Con = 8, AD-Ctsb = 11), Cortex (CTX); Dentate gyrus (DG).

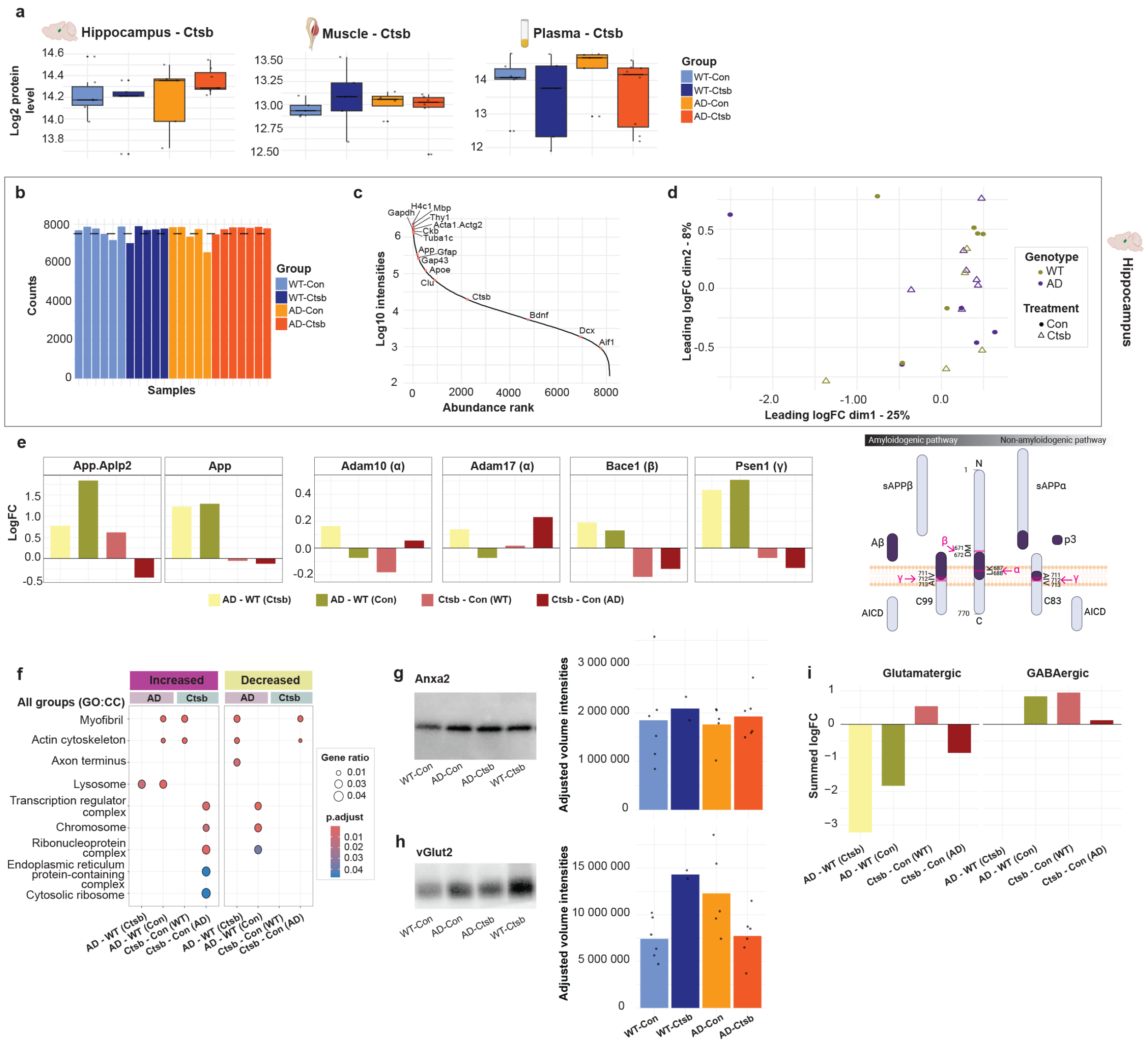

Fig. S5. Additional proteomics data for hippocampus including quality control and gene set enrichment analyses. (a) Protein abundance of Ctsb in hippocampus, muscle and plasma across all experimental groups. (b) 7509 proteins were reliably quantified in the hippocampus. (c) Dynamic range highlighting abundance of relevant proteins in AD pathogenesis. (d) Multidimensional scaling did not show any clustering of samples. (e) LogFC of App and the secretases involved in its processing across groups. (f) GSEA of the hippocampus, summarizing enriched cellular component (CC) ontologies in all groups. (g-h) Western blot of ANXA2 and vGLUT2 levels (WT-Con, N=6; WT-Ctsb, N=2; AD-Con, N=5; AD-Ctsb, N=6). (i) Summed logFC intensities of all proteins involved in glutamatergic (GO: 0035249, 56 proteins) and GABAergic (GO: 0051932, 37 proteins) synaptic transmission. Data for pairwise comparisons are annotated as follows: AD-WT(Ctsb), AD-Ctsb vs WT-Ctsb; AD-WT(Con), AD-Con vs WT-Con; Ctsb-Con(WT), WT-Ctsb vs WT-Con; Ctsb-Con(AD), AD-Ctsb vs AD-Con.

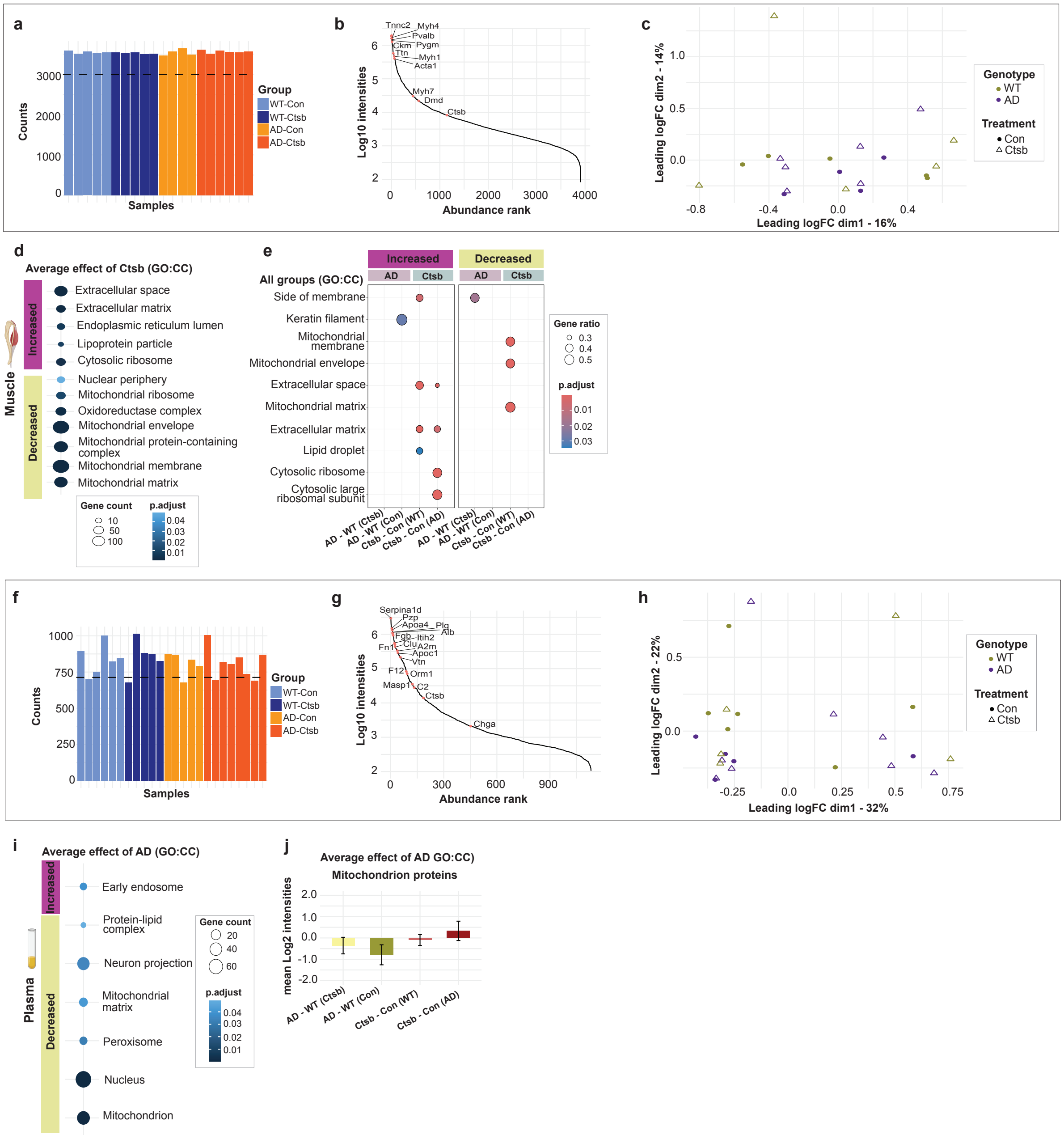

Fig. S6. Additional proteomics data from muscle and plasma, including quality control and gene set enrichment analyses. (a) 2998 proteins were quantified in the muscle after filtering. **(b)** Dynamic range highlighting abundance of classical muscle proteins. **(c)** Multidimensional scaling did not show any clustering of samples. **(d-e)** GSEA of the muscle, showing enriched CC ontologies **(d)** by average effect of Ctsb treatment and **(e)** across all groups. **(f)** 713 proteins were quantified in the plasma after filtering. **(g)** Dynamic range plasma proteins. **(h)** Multidimensional scaling did not show any clustering of samples. **(i)** GSEA of the plasma, showing enriched CC terms based on the average effect of AD. **(j)** Mean abundance of proteins related to mitochondrion showed a tendency towards increase only in AD-Ctsb plasma. For

GSEAs, a cut-off of FDR < 0.05 was used **(d, e, i, j).** Data for pairwise comparisons are annotated as follows: AD-WT(Ctsb), *AD-Ctsb vs WT-Ctsb*; AD-WT(Con), *AD-Con vs WT-Con*; Ctsb-Con(WT), *WT-Ctsb vs WT-Con*; Ctsb-Con(AD), *AD-Ctsb vs AD-Con*.

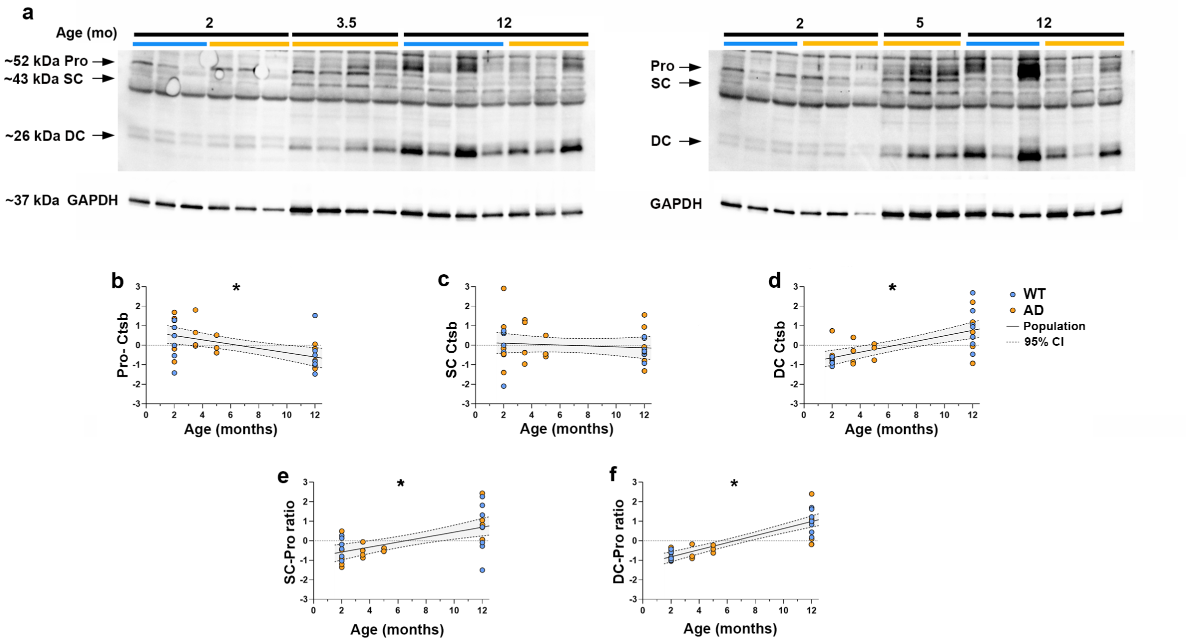

Fig. S7. Muscle Ctsb levels increase with aging. Additional cohorts of mice were utilized to monitor Ctsb levels with aging. **(a)** Western blotting (WB) of muscle Ctsb levels in two month (WT, N=6; AD, N=6), three and a half month (AD-Con, N=4), five month (AD-Con, N=3) and twelve month old mice (WT-Con, N=7, AD-Con, N=6), showed **(b)** a decrease in pro-Ctsb (Pro), **(c)** no change in single chain (SC), **(d)** an increase in double-chain (DC); and in ratios of **(e)** single- and **(f)** double-chain Ctsb to pro-Ctsb, with increasing age. Solid lines represent effect of age Ctsb levels while dashed lines represent the 95% CI. For this analysis two WB membranes were used. Each membrane contained samples from both genotypes with age ranging from 2-12 months old. Before the analysis was run, all values from the same membrane were first normalized to Z-scores using the average and standard deviation values of that membrane. The normalized values from both membranes were used in the GLM analysis (Tables S29-S33). *P<0.05 for effect of age.

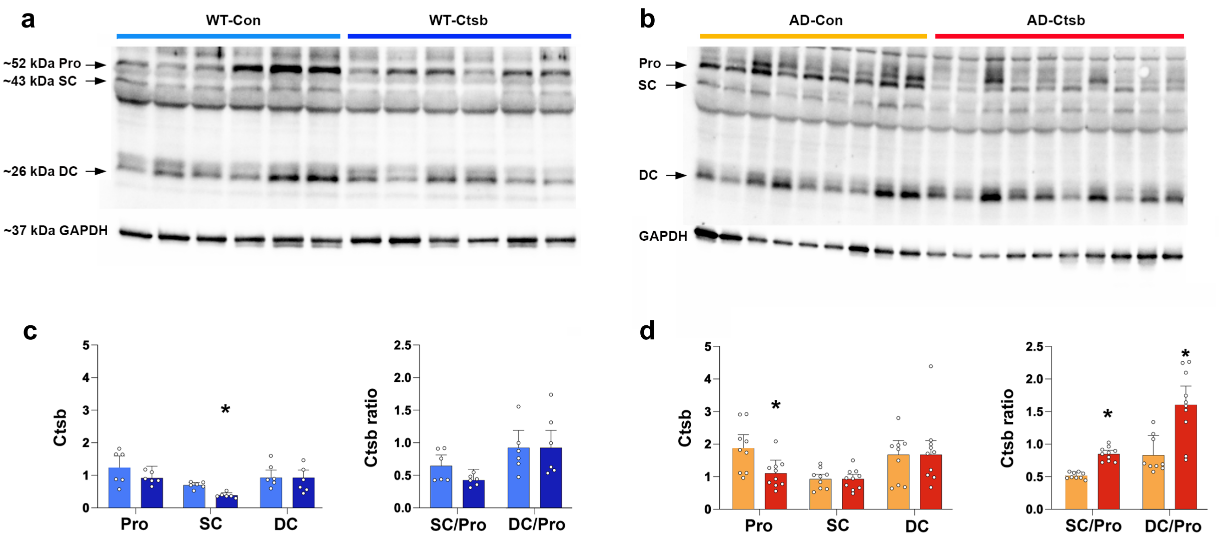

Fig. S8. Muscle Ctsb processing is modified by vector injection. Gastrocnemius muscle derived from **(a)** WT mice (WT-Con, N=6; WT-Ctsb, N=6), eight weeks after vector injection and **(b)** AD mice (AD-Con, N=9; AD-Ctsb, N=10) six to eight weeks after vector injection showed differential changes in immature (Pro) and mature (single-chain (SC) and double-chain (DC)) Ctsb protein. **(c)** In WT-Ctsb muscle SC protein is reduced as compared to WT-Con. **(d)** In AD-Ctsb muscle, pro-Ctsb is reduced, and the ratios of SC and DC to pro-Ctsb are increased as compared to AD-Con muscle. Data were analyzed by GLM (Tables S34-S43). $*P<0.05$

Table S1. Summary of the selected model for analysis of total distance traveled during 60 min activity box session

**Method**	Generalized linear model (GLM)				
**Family distribution with link function**	Gamma (log)				
**Formula**	Total distance traveled ~ Genotype * Treatment + Latency to fall				
**Fixed effects**		**β**	**SE**	**t value**	**Pr(>|t|)**
	Intercept	8.1152	0.2479	32.740	<2e-16 ***
	GenAD	0.3103	0.2266	1.370	0.1793
	TreatCTSB	0.3522	0.1707	2.063	0.0464 *
	Latency to fall	0.5995	0.4218	1.421	0.1639
	GenAD:TreatCTSB	-0.5297	0.2811	-1.884	0.0676
**Diagnosis**	No diagnosis problems detected				
**Other information**	Dispersion parameter for Gamma family taken to be 0.1313971<br>Null deviance: 5.4227 on 40 degrees of freedom<br>Residual deviance: 4.7259 on 36 degrees of freedom				

β = estimated coefficients for the fixed effect terms; SE = Standard Error; Intercept = WT-CON; Significance codes: *** 0.001; ** 0.01; * 0.05

Table S2. Summary of the selected model for analysis of distance traveled over time (20 min bins) during 60 min activity box session

<table>
<tr><td>Method</td><td colspan="5">Generalized linear mixed model (GLMM)</td></tr>
<tr><td>Family distribution with link function</td><td colspan="5">Gamma (log)</td></tr>
<tr><td>Formula</td><td colspan="5">Distance traveled ~ Time + (1 | id)</td></tr>
<tr><td>Random effects</td><td></td><td>Variance</td><td>SD</td><td></td><td></td></tr>
<tr><td></td><td>id</td><td>0.09638</td><td>0.3104</td><td></td><td></td></tr>
<tr><td># of observations</td><td>123</td><td></td><td></td><td></td><td></td></tr>
<tr><td># of id</td><td>41</td><td></td><td></td><td></td><td></td></tr>
<tr><td>Fixed effects</td><td></td><td>β</td><td>SE</td><td>z value</td><td>Pr(>|z|)</td></tr>
<tr><td></td><td>Intercept</td><td>8.1621</td><td>0.1481</td><td>55.09</td><td>< 2e-16 ***</td></tr>
<tr><td></td><td>Bin2</td><td>-0.9795</td><td>0.1987</td><td>-4.93</td><td>8.23e-07 ***</td></tr>
<tr><td></td><td>Bin3</td><td>-2.3121</td><td>0.2172</td><td>-10.64</td><td><2e-16 ***</td></tr>
<tr><td>Diagnosis</td><td colspan="5">Residuals normality problems detected;
Residuals dispersion problems detected;
Outliers detected;
Within-group deviations from uniformity detected;</td></tr>
<tr><td>Other information</td><td colspan="5">Before starting the analysis, the data were corrected for the use of Gamma distribution through the following procedure:
A positive constant (epsilon = 0.000001) was add to each individual value

Models ranked by AICc (dredge function from the MuMin package)
Dispersion parameter for Gamma family taken to be 0.782

Estimated Marginal Means and their respective 95% Confidence Intervals
(values are presented as cm)

Time:
Bin 1 = 3505; 2614 – 4701
Bin 2 = 1316; 981 – 1766 [a]
Bin 3 = 347; 250 – 482 [a,b]</td></tr>
</table>

SD = Standard Deviation; β = estimated coefficients for the fixed effect terms; SE = Standard Error; Intercept = Bin 1; [a] indicates p= 0.0001 when compared to Bin 1; [b] indicates p= 0.0001 when compared to Bin 2; Significance codes: *** 0.001; ** 0.01; * 0.05

Table S3: Summary of the selected model for analysis of latency to fall in the rotarod test

**Method**	Generalized linear model (GLM)				
**Family distribution with link function**	Beta (logit)				
**Formula**	Latency to fall ~ Genotype * Treatment				
**Fixed effects**		**β**	**SE**	**z value**	**Pr(>|z|)**
	Intercept	0.07165	0.17334	0.413	0.679
	GenAD	-1.57622	0.29597	-5.326	1.01e-07 ***
	TreatCTSB	-0.34508	0.25288	-1.365	0.172
	GenAD:TreatCTSB	1.70036	0.37839	4.494	7.00e-06 ***
**Diagnosis**	No diagnosis problems detected				
**Other information**	Before starting the analysis, the data were corrected for the use of Beta distribution through the following procedure: Each latency to fall value (y) was obtaining as a frequency of the total duration of the test (i.e., 0 < y < 1), as follows: Latency to fall (s) / 300 (s); Dispersion parameter for Beta family taken to be 12.3				

β = estimated coefficients for the fixed effect terms; SE = Standard Error; Intercept = WT-CON; Significance codes: *** 0.001; ** 0.01; * 0.05

Table S4: Summary of the selected model for analysis of platform latency during the acquisition phase (4-day bins) of the Morris water maze paradigm

**Method**	Generalized linear mixed model (GLMM)				
**Family distribution with link function**	Beta (logit)				
**Formula**	Latency to find the platform ~ Genotype * Treatment * Time + (1 | id)				
**Random effects**		**Variance**	**SD**		
	id	0.5702	0.7551		
**# of observations**	205				
**# of id**	41				
**Fixed effects**		**β**	**SE**	**z value**	**Pr(>|z|)**
	Intercept	2.65612	0.38324	6.931	4.19e-12 ***
	GenAD	1.48835	0.60539	2.458	0.013953 *
	TreatCTSB	-0.14407	0.54371	-0.265	0.791021
	Bin2	-2.42801	0.35810	-6.780	1.20e-11 ***
	Bin3	-3.43976	0.36732	-9.365	< 2e-16 ***
	Bin4	-3.29858	0.36579	-9.018	< 2e-16 ***
	Bin5	-3.97430	0.37888	-10.490	< 2e-16 ***
	GenAD:TreatCTSB	-1.17818	0.78560	-1.500	0.133685
	GenAD:Bin2	-1.03438	0.57398	-1.802	0.071527
	GenAD:Bin3	0.29789	0.58088	0.513	0.608078
	GenAD:Bin4	-0.65492	0.57451	-1.140	0.254302
	GenAD:Bin5	-0.61197	0.59110	-1.035	0.300525
	TreatCTSB:TimeBin2	-0.16939	0.50542	-0.335	0.737518
	TreatCTSB:TimeBin3	0.10379	0.51927	0.200	0.841583
	TreatCTSB:TimeBin4	0.27863	0.51889	0.537	0.591286
	TreatCTSB:TimeBin5	-0.01328	0.53588	-0.025	0.980225
	GenAD:TreatCTSB:TimeBin2	2.96463	0.76328	3.884	0.00010 ***
	GenAD:TreatCTSB:TimeBin3	0.12509	0.74823	0.167	0.867230
	GenAD:TreatCTSB:TimeBin4	1.05775	0.75049	1.409	0.158713
	GenAD:TreatCTSB:TimeBin5	1.45907	0.76984	1.895	0.058053
**Diagnosis**	Residuals normality problems detected;<br>Residuals dispersion problems detected;<br>Quantile deviations detected;				
**Other information**	Before starting the analysis, the data were corrected for the use of Beta distribution through the following procedure:<br>For each 4-day bin, each latency to find the platform value (y) was obtaining as a frequency of the total duration of the test (i.e., 0 < y < 1), as follows:<br>Latency to find the platform (s) / 60 (s);<br>Later, a negative constant (epsilon = -0.000001) was add to each individual value<br><br>Dispersion parameter for Beta family taken to be 9.67				

SD = Standard Deviation; β = estimated coefficients for the fixed effect terms; SE = Standard Error; Intercept = WT-CON Bin 1; Significance codes: *** 0.001; ** 0.01; * 0.05

Table S5: Summary of the selected model for analysis of average speed during day 1 of the acquisition phase of the Morris water maze paradigm

**Method**	Generalized linear model (GLM)				
**Family distribution with link function**	Gamma (log)				
**Formula**	Average speed day 1 ~ 1				
**Fixed effects**		**β**	**SE**	**t value**	**Pr(>|t|)**
	Intercept	2.43768	0.02302	105.9	<2e-16 ***
**Diagnosis**	No diagnosis problems detected				
**Other information**	Dispersion parameter for Gamma family taken to be 0.02172645<br>Null deviance: 0.91201 on 40 degrees of freedom<br>Residual deviance: 0.91201 on 40 degrees of freedom<br><br>Estimated Marginal Means and their respective 95% Confidence Intervals for the full model, i.e., Average speed day 1 ~ Genotype * Treatment<br>(values are presented as cm/s)<br><br>Treatment = CON:<br>WT = 11.4; 10.4 – 12.6<br>AD = 11.1; 9.96 – 12.4<br><br>Treatment = CTSB:<br>WT = 12.2; 11.0 – 13.5<br>AD = 11.2; 10.32 – 12.1				

β = estimated coefficients for the fixed effect terms; SE = Standard Error; Intercept = Overall; Significance codes: *** 0.001; ** 0.01; * 0.05

Table S6: Summary of the selected model for analysis of average speed during the acquisition phase (4-day bins) of the Morris water maze

**Method**	Generalized linear mixed model (GLMM) - Linear mixed model fit by maximum likelihood ['lmerMod']			
**Family distribution with link function**	Gaussian (identity)			
**Formula**	Average speed ~ 1 + (1 | id)			
**Random effects**		**Variance**	**SD**	
	id	0.03044	0.1745	
	Residual	0.56794	0.7536	
**# of observations**	205			
**# of id**	41			
**Fixed effects**		**β**	**SE**	**t value**
	Intercept	11.33932	0.05927	191.3
**Diagnosis**	No diagnosis problems detected			
**Other information**	GLMM did not run when using Gamma distribution (log). Because the variable is continuous and present no values near to the lower boundary, the model was run using Gaussian distribution (identity).<br><br>Models ranked by AICc (dredge function from the MuMin package)			

SD = Standard Deviation; β = estimated coefficients for the fixed effect terms; SE = Standard Error; Intercept = Overall; Significance codes: *** 0.001; ** 0.01; * 0.05

Table S7: Summary of the selected model for analysis of time spent in the target quadrant during the 4h probe trial in the Morris water maze

**Method**	Generalized linear model (GLM)				
**Family distribution with link function**	Beta (logit)				
**Formula**	Time spent in the target quadrant ~ Genotype + Treatment + Speed + Genotype:Treatment + Treatment:Speed				
**Fixed effects**		**β**	**SE**	**z value**	**Pr(>|z|)**
	Intercept	-0.069466	0.690288	-0.101	0.919841
	GenAD	-0.755924	0.227027	-3.330	0.000869 ***
	TreatCTSB	-3.772909	1.167028	-3.233	0.001225 **
	Speed	-0.005502	0.059120	-0.093	0.925856
	GenAD:TreatCTSB	1.256673	0.317114	3.963	7.41e-05 ***
	TreatCTSB:Speed	0.239705	0.095948	2.498	0.012480 *
**Diagnosis**	No diagnosis problems detected				
**Other information**	Before starting the analysis, the data were corrected for the use of Beta distribution through the following procedure:<br>Each time spent in the target quadrant (y) was obtaining as a frequency of the total duration of the test (i.e., 0 < y < 1), as follows:<br>Time spent in the target quadrant (s) / 60 (s);<br><br>Dispersion parameter for Beta family taken to be 18.5<br><br>Estimated Marginal Means and their respective 95% Confidence Intervals when Speed is fixed at 11.4 cm/s (the overall average speed during day 1)<br>(values are presented as %)<br><br>Treatment = CON:<br>WT = 46.7; 39.6 – 54.0<br>AD = 29.1; 22.3 – 37.1<br><br>Treatment = CTSB:<br>WT = 23.8; 17.9 – 31.0<br>AD = 34.1; 28.4 – 40.2				

Speed = Average speed during day 1 of the acquisition phase; β = estimated coefficients for the fixed effect terms; SE = Standard Error; Intercept = WT-CON; Significance codes: *** 0.001; ** 0.01; * 0.05

Table S8: Summary of the one-sample Wilcoxon signed rank test for analysis of time spent in the target quadrant during the 4h probe trial in the Morris water maze paradigm against the 25% chance

**Method**	One-sample Wilcoxon signed rank exact test (one-tail test)				
**Reference value**	25% (mu = 0.25)				
**Alternative Hypothesis**	one-tail test where the H1 = location is greater than 25% chance				
**Output**		**V value**	**N**	**p-value**	**one-tail**
	WT-CON	55	10	0.000977	greater
	WT-CTSB	26	9	0.3672	equal
	AD-CON	25	8	0.1914	equal
	AD-CTSB	84	14	0.02472	greater

N= sample size

Table S9: Summary of the selected model for analysis of time spent in the target quadrant during the 24h probe trial in the Morris water maze

**Method**	Generalized linear model (GLM)				
**Family distribution with link function**	Beta (logit)				
**Formula**	Time spent in the target quadrant ~ Genotype * Treatment * Speed				
**Fixed effects**		**β**	**SE**	**z value**	**Pr(>|z|)**
	Intercept	-0.72510	0.91886	-0.789	0.430038
	GenAD	-4.00382	1.41351	-2.833	0.004618 **
	TreatCTSB	-4.54110	2.10162	-2.161	0.030714 *
	Speed	0.02693	0.07961	0.338	0.735174
	GenAD:TreatCTSB	8.64317	2.51605	3.435	0.000592 ***
	GenAD:Speed	0.25822	0.12030	2.146	0.031842 *
	TreatCTSB:Speed	0.32570	0.17222	1.891	0.058601
	GenAD:TreatCTSB:Speed	-0.62992	0.20913	-3.012	0.002595 **
**Diagnosis**	No diagnosis problems detected				
**Other information**	Before starting the analysis, the data were corrected for the use of Beta distribution through the following procedure:<br>Each time spent in the target quadrant (y) was obtaining as a frequency of the total duration of the test (i.e., 0 < y < 1), as follows:<br>Time spent in the target quadrant (s) / 60 (s);<br><br>Dispersion parameter for Beta family taken to be 22.2<br><br>Estimated Marginal Means and their respective 95% Confidence Intervals when Speed is fixed at 11.4 cm/s (the overall average speed during day 1)<br>(values are presented as %)<br><br>Treatment = CON:<br>WT = 39.7; 33.4 – 46.4<br>AD = 18.8; 13.6 – 25.3<br><br>Treatment = CTSB:<br>WT = 22.6; 16.1 – 30.8<br>AD = 30.0; 25.1 – 35.5				

Speed = Average speed during day 1 of the acquisition phase; β = estimated coefficients for the fixed effect terms; SE = Standard Error; Intercept = WT-CON; Significance codes: *** 0.001; ** 0.01; * 0.05

Table S10: Summary of the one-sample Wilcoxon signed rank test for analysis of time spent in the target quadrant during the 24h probe trial in the Morris water maze against the 25% chance

**Method**	One-sample Wilcoxon signed rank exact test (one-tail test)				
**Reference value**	25% (mu = 0.25)				
**Alternative Hypothesis**	one-tail test where the H1 = location is greater than 25% chance				
**Output**		**V value**	**N**	**p-value**	**one-tail**
	WT-CON	55	10	0.000977	greater
	WT-CTSB	29	9	0.248	equal
	AD-CON	14	8	0.7266	equal
	AD-CTSB	81	14	0.03925	greater

N= sample size

Table S11: Summary of the selected model for analysis of percentage of freezing during the conditioning phase of the fear conditioning paradigm

**Method**	Generalized linear mixed model (GLMM)				
**Family distribution with link function**	Beta (logit)				
**Formula**	Freezing behavior ~ Genotype * Treatment * Tone-Shock + (1 | id)				
**Random effects**		**Variance**	**SD**		
	id	0.2987	0.5465		
**# of observations**	123				
**# of id**	41				
**Fixed effects**		**β**	**SE**	**z value**	**Pr(>|z|)**
	Intercept	-0.6223	0.3731	-1.668	0.09532
	GenAD	0.4815	0.5490	0.877	0.38044
	TreatCTSB	-0.4615	0.5412	-0.853	0.39380
	TS2	1.1367	0.4559	2.493	0.01265 *
	TS3	1.5231	0.4631	3.288	0.00101 **
	GenAD:TreatCTSB	0.1192	0.7427	0.160	0.87250
	GenAD:TS2	-0.7094	0.6700	-1.059	0.28969
	GenAD:TS3	-0.6574	0.6675	-0.985	0.32470
	TreatCTSB:TimeTS2	-0.8111	0.6592	-1.230	0.21854
	TreatCTSB:TimeTS3	-0.8471	0.6519	-1.299	0.19382
	GenAD:TreatCTSB:TimeTS2	0.8975	0.9062	0.990	0.32198
	GenAD:TreatCTSB:TimeTS3	1.1078	0.8938	1.239	0.21522
**Diagnosis**	No diagnosis problems detected				
**Other information**	Before starting the analysis, the data were corrected for the use of Beta distribution through the following procedure:<br>For each Tone-Shock presentation, each freezing behavior value (y) was obtaining as a frequency of the total duration of each tone presentation (i.e., 0 < y < 1), as follows:<br>Freezing behavior (s) / 30 (s);<br><br>Dispersion parameter for Beta family taken to be 3.18				

SD = Standard Deviation; β = estimated coefficients for the fixed effect terms; SE = Standard Error; Intercept = WT-CON Tone-Shock presentation 1; TS = Tone-Shock presentation; Significance codes: *** 0.001; ** 0.01; * 0.05

Table S12: Summary of the selected model for analysis of percentage of freezing during the tone-cued phase of the fear conditioning paradigm

**Method**	Generalized linear model (GLM)				
**Family distribution with link function**	Beta (logit)				
**Formula**	Freezing behavior ~ Genotype + Treatment + TS1 + Genotype:Treatment + Genotype:TS1 + Treatment:TS1				
**Fixed effects**		**β**	**SE**	**z value**	**Pr(>|z|)**
	Intercept	0.606080	0.750491	0.808	0.4193
	GenAD	-1.287511	0.954155	-1.349	0.1772
	TreatCTSB	0.003667	0.795825	0.005	0.9963
	TS1	0.009905	0.021068	0.470	0.6382
	GenAD:TreatCTSB	1.267221	0.813075	1.559	0.1191
	GenAD:TS1	0.033867	0.020073	1.687	0.0916
	TreatCTSB:TS1	-0.034950	0.020324	-1.720	0.0855
**Diagnosis**	No diagnosis problems detected				
**Other information**	Before starting the analysis, the data were corrected for the use of Beta distribution through the following procedure: For each Tone presentation (T1-T3), each freezing behavior value (y) was obtaining as a frequency of the total duration of each tone presentation (i.e., $0 < y < 1$), as follows: Freezing behavior (s) / 30 (s); After that, the average frequency for T1-T3 was calculated. Later, a negative constant (epsilon = -0.000001) was add to each individual value. Dispersion parameter for Beta family taken to be 1.8				

SD = Standard Deviation; β = estimated coefficients for the fixed effect terms; SE = Standard Error; Intercept = WT-CON Tone presentation 1; TS1 = First Tone-Shock presentation during the conditioning phase; T = Tone presentation; Significance codes: *** 0.001; ** 0.01; * 0.05

Table S13: Summary of the selected model for analysis of percentage of freezing during the contextual phase of the fear conditioning paradigm

**Method**	Generalized linear mixed model (GLMM)				
**Family distribution with link function**	Beta (logit)				
**Formula**	Freezing behavior ~ Genotype * Treatment * Session + (1 | id)				
**Random effects**		**Variance**	**SD**		
	id	0.5312	0.7288		
**# of observations**	164				
**# of id**	41				
**Fixed effects**		**β**	**SE**	**z value**	**Pr(>|z|)**
	Intercept	-1.93143	0.38417	-5.028	4.97e-07 ***
	GenAD	-0.30933	0.59103	-0.523	0.60071
	TreatCTSB	-0.28857	0.56778	-0.508	0.61129
	Session2	0.55537	0.40801	1.361	0.17346
	Session3	0.69847	0.40197	1.738	0.08228
	Session4	0.48018	0.40974	1.172	0.24124
	GenAD:TreatCTSB	0.54790	0.79966	0.685	0.49324
	GenAD:Session2	2.70447	0.62860	4.302	1.69e-05 ***
	GenAD:Session3	1.54914	0.60497	2.561	0.01045 *
	GenAD:Session4	1.47637	0.63775	2.315	0.02062 *
	TreatCTSB:Session2	0.05153	0.60969	0.085	0.93264
	TreatCTSB:Session3	-0.04091	0.60177	-0.068	0.94579
	TreatCTSB:Session4	0.23679	0.61103	0.388	0.69837
	GenAD:TreatCTSB:Session2	-2.45644	0.85187	-2.884	0.00393 **
	GenAD:TreatCTSB:Session3	-1.02730	0.82343	-1.248	0.21218
	GenAD:TreatCTSB:Session4	-1.04578	0.85272	-1.226	0.22005
**Diagnosis**	Residuals normality problems detected;<br>Quantile deviations detected;				
**Other information**	Before starting the analysis, the data were corrected for the use of Beta distribution through the following procedure:<br>For each Session, each freezing behavior value (y) was obtaining as a frequency of the total duration of the session (i.e., 0 < y < 1), as follows:<br>Freezing behavior (s) / 330 (s);<br><br>Dispersion parameter for Beta family taken to be 5.7				

SD = Standard Deviation; β = estimated coefficients for the fixed effect terms; SE = Standard Error; Intercept = WT-CON Session 1; Significance codes: *** 0.001; ** 0.01; * 0.05

Table S14: Summary of the selected model for analysis of mean number of DCX$^+$ cells in the dentate gyrus of the hippocampus

**Method**	Generalized linear model (GLM)				
**Family distribution with link function**	Gamma (log)				
**Formula**	DCX+ cells ~ Genotype * Treatment				
**Fixed effects**		**β**	**SE**	**t value**	**Pr(>|t|)**
	Intercept	2.3023	0.1329	17.321	< 2e-16 ***
	GenAD	-0.6702	0.1994	-3.362	0. 001886 **
	TreatCTSB	-0. 3672	0. 1994	-1. 842	0. 074014
	GenAD:TreatCTSB	1.0129	0. 2746	3.688	0. 000761 ***
**Diagnosis**	No diagnosis problems detected				
**Other information**	Dispersion parameter for Gamma family taken to be 0.1766747<br>Null deviance: 8.4771 on 38 degrees of freedom<br>Residual deviance: 5.8968 on 35 degrees of freedom				

β = estimated coefficients for the fixed effect terms; SE = Standard Error; Intercept = WT-CON; Significance codes: *** 0.001; ** 0.01; * 0.05

Table S15: Summary of the selected model for analysis of mean number of BrdU$^+$ cells in the dentate gyrus of the hippocampus

**Method**	Generalized linear model (GLM)				
**Family distribution with link function**	Gamma (log)				
**Formula**	BrdU+ cells ~ 1				
**Fixed effects**		**β**	**SE**	**t value**	**Pr(>|t|)**
	Intercept	0.1248	0.1502	0.831	0.411
**Diagnosis**	Outlier detected;<br>No other diagnosis problems detected				
**Other information**	Dispersion parameter for Gamma family taken to be 0.9019985<br>Null deviance: 110.23 on 39 degrees of freedom<br>Residual deviance: 110.23 on 39 degrees of freedom<br><br>Estimated Marginal Means and their respective 95% Confidence Intervals (values are presented as number of cells)<br><br>Population = 1.13; 0.84 – 1.54				

β = estimated coefficients for the fixed effect terms; SE = Standard Error; Intercept = Overall; Significance codes: *** 0.001; ** 0.01; * 0.05

Table S16: Summary of the Spearman's rank correlation rho test for analysis of correlation between DCX$^+$ cells and BrdU$^+$ cells

**Method**	Spearman's rank correlation rho				
**Alternative Hypothesis**	H1 = true rho (ρ) is not equal to 0				
**Output**		**S value**	**N**	**rho (ρ)**	**p-value**
	Population	5131	39	0.481	0.0020 *
	WT-CON	64	10	0.612	0.0665
	WT-CTSB	52.12	8	0.380	0.3538
	AD-CON	88.024	8	-0.048	0.9103
	AD-CTSB	126	13	0.654	0.0183 *

N= sample size; rho (ρ) = Spearman correlation coefficient; Significance codes: *** 0.001; ** 0.01; * 0.05

Table S17: Summary of the selected model for analysis of density of ThioS$^+$ labeling in the cortex

**Method**	Generalized linear model (GLM)				
**Family distribution with link function**	Beta (logit)				
**Formula**	Density of ThioS ~ 1				
**Fixed effects**		**β**	**SE**	**z value**	**Pr(>|z|)**
	Intercept	-3.6698	0.1639	-22.38	<2e-16 ***
**Diagnosis**	No diagnosis problems detected				
**Other information**	Before starting the analysis, the data were corrected for the use of Beta distribution through the following procedure:<br>Each percentage of ThioS+ area value (y) was obtaining as a frequency value (i.e., 0 < y < 1), as follows:<br>ThioS+ area (%) / 100;<br><br>Dispersion parameter for Beta family taken to be 79.8<br><br>Estimated Marginal Means and their respective 95% Confidence Intervals (values are presented as % of area)<br><br>Population = 2.48; 1.77 – 3.48				

β = estimated coefficients for the fixed effect terms; SE = Standard Error; Intercept = Overall; Significance codes: *** 0.001; ** 0.01; * 0.05

Table S18: Summary of the selected model for analysis of hippocampal ThioS^{+} labeling density

**Method**	Generalized linear model (GLM)				
**Family distribution with link function**	Beta (logit)				
**Formula**	Density of ThioS ~ 1				
**Fixed effects**		**β**	**SE**	**z value**	**Pr(>|z|)**
	Intercept	-4.0747	0.2058	-19.8	<2e-16 ***
**Diagnosis**	No diagnosis problems detected				
**Other information**	Before starting the analysis, the data were corrected for the use of Beta distribution through the following procedure:<br>Each percentage of ThioS+ area value (y) was obtaining as a frequency value (i.e., 0 < y < 1), as follows:<br>ThioS+ area (%) / 100;<br><br>Dispersion parameter for Beta family taken to be 74.6<br><br>Estimated Marginal Means and their respective 95% Confidence Intervals (values are presented as % of area)<br><br>Population = 1.67; 1.09 – 2.56				

β = estimated coefficients for the fixed effect terms; SE = Standard Error; Intercept = Overall; Significance codes: *** 0.001; ** 0.01; * 0.05

Table S19: Summary of the selected model for analysis of ThioS+ plaque number in the cortex

**Method**	Generalized linear model (GLM)				
**Family distribution with link function**	Gamma (log)				
**Formula**	Number of ThioS ~ 1				
**Fixed effects**		**β**	**SE**	**z value**	**Pr(>|z|)**
	Intercept	4.0934	0.1336	30.63	<2e-16 ***
**Diagnosis**	No diagnosis problems detected				
**Other information**	Dispersion parameter for Gamma family taken to be 0.339<br><br>Estimated Marginal Means and their respective 95% Confidence Intervals (values are presented as % of area)<br><br>Population = 59.9; 45.2– 79.5				

β = estimated coefficients for the fixed effect terms; SE = Standard Error; Intercept = Overall; Significance codes: *** 0.001; ** 0.01; * 0.05

Table S20: Summary of the selected model for analysis of ThioS$^+$ plaque number in the hippocampus

**Method**	Generalized linear model (GLM)				
**Family distribution with link function**	Gamma (log)				
**Formula**	Number of ThioS ~ 1				
**Fixed effects**		**β**	**SE**	**z value**	**Pr(>|z|)**
	Intercept	3.8516	0.2132	18.06	<2e-16 ***
**Diagnosis**	No diagnosis problems detected				
**Other information**	Dispersion parameter for Gamma family taken to be 0.864<br><br>Estimated Marginal Means and their respective 95% Confidence Intervals (values are presented as % of area)<br><br>Population = 47.1; 30.0 – 73.8				

β = estimated coefficients for the fixed effect terms; SE = Standard Error; Intercept = Overall; Significance codes: *** 0.001; ** 0.01; * 0.05

Table S21: Summary of the selected model for analysis of hippocampal Iba1$^+$ labeling density

**Method**	Generalized linear model (GLM)				
**Family distribution with link function**	Beta (logit)				
**Formula**	Density of Iba1 ~ Genotype				
**Fixed effects**		**β**	**SE**	**z value**	**Pr(>|z|)**
	Intercept	-3.4050	0.1130	-30.121	<2e-16 ***
	GenAD	0.3612	0.1424	2.536	0.0112 *
**Diagnosis**	No diagnosis problems detected				
**Other information**	Before starting the analysis, the data were corrected for the use of Beta distribution through the following procedure:<br>Each percentage of Iba1+ area value (y) was obtaining as a frequency value (i.e., 0 < y < 1), as follows:<br>Iba1+ area (%) / 100;<br><br>Dispersion parameter for Beta family taken to be 131<br><br>Estimated Marginal Means and their respective 95% Confidence Intervals (values are presented as % of area)<br><br>WT = 3.21; 2.57 – 4.01<br>AD = 4.55; 3.80 – 5.43				

β = estimated coefficients for the fixed effect terms; SE = Standard Error; Intercept = WT; Significance codes: *** 0.001; ** 0.01; * 0.05

Table S22: Summary of the selected model for analysis of hippocampal GFAP$^+$ labeling density

**Method**	Generalized linear model (GLM)				
**Family distribution with link function**	Beta (logit)				
**Formula**	Density of GFAP ~ 1				
**Fixed effects**		**β**	**SE**	**z value**	**Pr(>|z|)**
	Intercept	-1.19702	0.07697	-15.55	<2e-16 ***
**Diagnosis**	No diagnosis problems detected				
**Other information**	Before starting the analysis, the data were corrected for the use of Beta distribution through the following procedure:<br>Each percentage of GFAP+ area value (y) was obtaining as a frequency value (i.e., 0 < y < 1), as follows:<br>GFAP+ area (%) / 100;<br><br>Dispersion parameter for Beta family taken to be 23.9<br><br>Estimated Marginal Means and their respective 95% Confidence Intervals (values are presented as % of area)<br><br>Population = 23.2; 20.5 – 26.1				

β = estimated coefficients for the fixed effect terms; SE = Standard Error; Intercept = Overall; Significance codes: *** 0.001; ** 0.01; * 0.05

Supplementary Tables S23-S28 are in Excel format.

Table S29: Summary of the selected model for analysis of muscle pro-Ctsb time-course

**Method**	Generalized linear model (GLM)				
**Family distribution with link function**	Gaussian (identity)				
**Formula**	pro-Ctsb expression ~ Age				
**Fixed effects**		**β**	**SE**	**t value**	**Pr(>|t|)**
	Intercept	0.71766	0.26147	2.745	0.01013 *
	Age	-0.10988	0.03272	-3.359	0.00214 **
**Diagnosis**	No diagnosis problems detected				
**Other information**	Two WB membranes were used in the analysis. Each membrane contained samples from both genotypes with age ranging from 2-12 months old.<br>Before analysis was ran, all values from the same membrane were first normalized to Z-score using the average and standard deviation values of that membrane. Each membrane was independently normalized to Z-scores and the normalized values from both membranes were used in the GLM analysis.<br><br>Dispersion parameter for Gaussian family taken to be 0.7267389<br><br>Null deviance: 30.000 on 31 degrees of freedom<br>Residual deviance: 21.802 on 30 degrees of freedom				

β = estimated coefficients for the fixed effect terms; SE = Standard Error; Intercept = Population Age at 0 months; Significance codes: *** 0.001; ** 0.01; * 0.05

Table S30: Summary of the selected model for analysis of muscle single chain-Ctsb time-course

**Method**	Generalized linear model (GLM)				
**Family distribution with link function**	Gaussian (identity)				
**Formula**	Single chain-Ctsb expression ~ 1				
**Fixed effects**		**β**	**SE**	**t value**	**Pr(>|t|)**
	Intercept	-1.570e-16	1.739e-01	0	1
**Diagnosis**	No diagnosis problems detected				
**Other information**	Two WB membranes were used in the analysis. Each membrane contained samples from both genotypes with age ranging from 2-12 months old.<br>Before analysis was ran, all values from the same membrane were first normalized to Z-score using the average and standard deviation values of that membrane. Each membrane was independently normalized to Z-scores and the normalized values from both membranes were used in the GLM analysis.<br><br>Dispersion parameter for Gaussian family taken to be 0.9677419<br><br>Null deviance: 30 on 31 degrees of freedom<br>Residual deviance: 30 on 31 degrees of freedom				

β = estimated coefficients for the fixed effect terms; SE = Standard Error; Intercept = Population; Significance codes: *** 0.001; ** 0.01; * 0.05

Table S31: Summary of the selected model for analysis of muscle double chain-Ctsb time-course

**Method**	Generalized linear model (GLM)				
**Family distribution with link function**	Gaussian (identity)				
**Formula**	double chain-Ctsb expression ~ Age				
**Fixed effects**		**β**	**SE**	**t value**	**Pr(>|t|)**
	Intercept	-0.9155	0.2286	-4.005	0.000376 ***
	Age	0.1402	0.0286	4.901	3.08e-05 ***
**Diagnosis**	No diagnosis problems detected				
**Other information**	Two WB membranes were used in the analysis. Each membrane contained samples from both genotypes with age ranging from 2-12 months old.<br>Before analysis was ran, all values from the same membrane were first normalized to Z-score using the average and standard deviation values of that membrane. Each membrane was independently normalized to Z-scores and the normalized values from both membranes were used in the GLM analysis.<br><br>Dispersion parameter for Gaussian family taken to be 0.5553363<br><br>Null deviance: 30.00 on 31 degrees of freedom<br>Residual deviance: 16.66 on 30 degrees of freedom				

β = estimated coefficients for the fixed effect terms; SE = Standard Error; Intercept = Population Age at 0 months; Significance codes: *** 0.001; ** 0.01; * 0.05

Table S32: Summary of the selected model for analysis of muscle single chain/pro-Ctsb ratio time-course

**Method**	Generalized linear model (GLM)				
**Family distribution with link function**	Gaussian (identity)				
**Formula**	single chain/pro-Ctsb ratio ~ Treatment				
**Fixed effects**		**β**	**SE**	**t value**	**Pr(>|t|)**
	Intercept	-0.83194	0.24399	-3.410	0.001875 **
	Age	0.12738	0.03053	4.172	0.000237 ***
**Diagnosis**	No diagnosis problems detected				
**Other information**	Two WB membranes were used in the analysis. Each membrane contained samples from both genotypes with age ranging from 2-12 months old.<br>Before analysis was ran, all values from the same membrane were first normalized to Z-score using the average and standard deviation values of that membrane. Each membrane was independently normalized to Z-scores and the normalized values from both membranes were used in the GLM analysis.<br><br>Dispersion parameter for Gaussian family taken to be 0.6327821<br><br>Null deviance: 30.000 on 31 degrees of freedom<br>Residual deviance: 18.983 on 30 degrees of freedom				

β = estimated coefficients for the fixed effect terms; SE = Standard Error; Intercept = Population Age at 0 months; Significance codes: *** 0.001; ** 0.01; * 0.05

Table S33: Summary of the selected model for analysis of muscle double chain/pro-Ctsb ratio time-course

**Method**	Generalized linear model (GLM)				
**Family distribution with link function**	Gaussian (identity)				
**Formula**	double chain/pro-Ctsb ratio ~ 1				
**Fixed effects**		**β**	**SE**	**t value**	**Pr(>|t|)**
	Intercept	-1.17986	0.15682	-7.523	2.18e-08 ***
	Age	0.18065	0.01962	9.206	3.03e-10 ***
**Diagnosis**	No diagnosis problems detected				
**Other information**	Two WB membranes were used in the analysis. Each membrane contained samples from both genotypes with age ranging from 2-12 months old.<br>Before analysis was ran, all values from the same membrane were first normalized to Z-score using the average and standard deviation values of that membrane. Each membrane was independently normalized to Z-scores and the normalized values from both membranes were used in the GLM analysis.<br><br>Dispersion parameter for Gaussian family taken to be 0.2614243<br><br>Null deviance: 30.0000 on 31 degrees of freedom<br>Residual deviance: 7.8427 on 30 degrees of freedom				

β = estimated coefficients for the fixed effect terms; SE = Standard Error; Intercept = Population Age at 0 months; Significance codes: *** 0.001; ** 0.01; * 0.05

Table S34: Summary of the selected model for analysis of muscle pro-Ctsb in WT mice

**Method**	Generalized linear model (GLM)				
**Family distribution with link function**	Gaussian (identity)				
**Formula**	pro-Ctsb expression ~ Treatment				
**Fixed effects**		**β**	**SE**	**t value**	**Pr(>|t|)**
	Intercept	1.2371	0.1609	7.687	1.67e-05***
	TreatCTSB	-0.3202	0.2276	-1.407	0.190
**Diagnosis**	No diagnosis problems detected<br>Model did not converge fully				
**Other information**	Dispersion parameter for Gaussian family taken to be 0.1554158<br><br>Null deviance: 1.8617 on 11 degrees of freedom<br>Residual deviance: 1.5542 on 10 degrees of freedom				

β = estimated coefficients for the fixed effect terms; SE = Standard Error; Intercept = WT-CON; Significance codes: *** 0.001; ** 0.01; * 0.05

Table S35: Summary of the selected model for analysis of muscle single chain-Ctsb in WT mice

**Method**	Generalized linear model (GLM)				
**Family distribution with link function**	Gaussian (identity)				
**Formula**	Single chain-Ctsb expression ~ Treatment				
**Fixed effects**		**β**	**SE**	**t value**	**Pr(>|t|)**
	Intercept	0.69801	0.03641	19.172	3.24e-09***
	TreatCTSB	-0.31433	0.05149	-6.105	0.000115 ***
**Diagnosis**	No diagnosis problems detected<br>Model did not converge fully				
**Other information**	Dispersion parameter for Gaussian family taken to be 0.007952886<br><br>Null deviance: 0.375945 on 11 degrees of freedom<br>Residual deviance: 0.079529 on 10 degrees of freedom				

β = estimated coefficients for the fixed effect terms; SE = Standard Error; Intercept = WT-CON; Significance codes: *** 0.001; ** 0.01; * 0.05

Table S36: Summary of the selected model for analysis of muscle double chain-Ctsb in WT mice

**Method**	Generalized linear model (GLM)				
**Family distribution with link function**	Gaussian (identity)				
**Formula**	double chain-Ctsb expression ~ 1				
**Fixed effects**		**β**	**SE**	**t value**	**Pr(>|t|)**
	Intercept	0.9272	0.1062	8.732	2.81e-06***
**Diagnosis**	No diagnosis problems detected				
**Other information**	Dispersion parameter for Gaussian family taken to be 0.135287<br><br>Null deviance: 1.4882 on 11 degrees of freedom<br>Residual deviance: 1.4882 on 11 degrees of freedom				

β = estimated coefficients for the fixed effect terms; SE = Standard Error; Intercept = Population; Significance codes: *** 0.001; ** 0.01; * 0.05

Table S37: Summary of the selected model for analysis of muscle single chain/pro-Ctsb ratio of WT mice

**Method**	Generalized linear model (GLM)				
**Family distribution with link function**	Gaussian (identity)				
**Formula**	single chain/pro-Ctsb ratio ~ Treatment				
**Fixed effects**		**β**	**SE**	**t value**	**Pr(>|t|)**
	Intercept	0.64686	0.07399	8.743	5.37e-06***
	TreatCTSB	-0.22002	0.10464	-2.103	0.0618
**Diagnosis**	No diagnosis problems detected<br>Model did not converge fully				
**Other information**	Dispersion parameter for Gaussian family taken to be 0.03284547<br><br>Null deviance: 0.47369 on 11 degrees of freedom<br>Residual deviance: 0.32845 on 10 degrees of freedom				

β = estimated coefficients for the fixed effect terms; SE = Standard Error; Intercept = WT-CON; Significance codes: *** 0.001; ** 0.01; * 0.05

Table S38: Summary of the selected model for analysis of muscle double chain/pro-Ctsb ratio in WT mice

**Method**	Generalized linear model (GLM)				
**Family distribution with link function**	Gaussian (identity)				
**Formula**	double chain/pro-Ctsb ratio ~ 1				
**Fixed effects**		**β**	**SE**	**t value**	**Pr(>|t|)**
	Intercept	0.9290	0.1196	7.768	8.63e-06***
**Diagnosis**	No diagnosis problems detected				
**Other information**	Dispersion parameter for Gaussian family taken to be 0.1716424<br><br>Null deviance: 1.8881 on 11 degrees of freedom<br>Residual deviance: 1.8881 on 11 degrees of freedom				

β = estimated coefficients for the fixed effect terms; SE = Standard Error; Intercept = Population; Significance codes: *** 0.001; ** 0.01; * 0.05

Table S39: Summary of the selected model for analysis of muscle pro-Ctsb in AD mice

**Method**	Generalized linear model (GLM)				
**Family distribution with link function**	Gaussian (identity)				
**Formula**	pro-Ctsb expression ~ Treatment				
**Fixed effects**		**β**	**SE**	**t value**	**Pr(>|t|)**
	Intercept	1.8708	0.1993	9.387	3.88e-08***
	TreatCTSB	-0.7644	0.2747	-2.782	0.0128 *
**Diagnosis**	No diagnosis problems detected<br>Model did not converge fully				
**Other information**	Dispersion parameter for Gaussian family taken to be 0.3574917<br><br>Null deviance: 8.8448 on 18 degrees of freedom<br>Residual deviance: 6.0774 on 17 degrees of freedom				

β = estimated coefficients for the fixed effect terms; SE = Standard Error; Intercept = AD-CON; Significance codes: *** 0.001; ** 0.01; * 0.05

Table S40: Summary of the selected model for analysis of muscle single chain-Ctsb in AD mice

**Method**	Generalized linear model (GLM)				
**Family distribution with link function**	Gaussian (identity)				
**Formula**	Single chain-Ctsb expression ~ 1				
**Fixed effects**		**β**	**SE**	**t value**	**Pr(>|t|)**
	Intercept	0.92977	0.06838	13.6	6.59e-11***
**Diagnosis**	No diagnosis problems detected				
**Other information**	Dispersion parameter for Gaussian family taken to be 0.08884275<br><br>Null deviance: 1.5992 on 18 degrees of freedom<br>Residual deviance: 1.5992 on 18 degrees of freedom				

β = estimated coefficients for the fixed effect terms; SE = Standard Error; Intercept = Population; Significance codes: *** 0.001; ** 0.01; * 0.05

Table S41: Summary of the selected model for analysis of muscle double chain-Ctsb in AD mice

**Method**	Generalized linear model (GLM)				
**Family distribution with link function**	Gaussian (identity)				
**Formula**	double chain-Ctsb expression ~ 1				
**Fixed effects**		**β**	**SE**	**t value**	**Pr(>|t|)**
	Intercept	1.6790	0.2048	8.196	1.73e-07***
**Diagnosis**	No diagnosis problems detected<br>Model did not converge fully				
**Other information**	Dispersion parameter for Gaussian family taken to be 0.7972739<br><br>Null deviance: 14.351 on 18 degrees of freedom<br>Residual deviance: 14.351 on 18 degrees of freedom				

β = estimated coefficients for the fixed effect terms; SE = Standard Error; Intercept = Population; Significance codes: *** 0.001; ** 0.01; * 0.05

Table S42: Summary of the selected model for analysis of muscle single chain/pro-Ctsb ratio in AD mice

**Method**	Generalized linear model (GLM)				
**Family distribution with link function**	Gaussian (identity)				
**Formula**	single chain/pro-Ctsb ratio ~ Treatment				
**Fixed effects**		**β**	**SE**	**t value**	**Pr(>|t|)**
	Intercept	0.51639	0.02777	18.596	9.80e-13 ***
	TreatCTSB	0.33359	0.03828	8.715	1.12e-07 ***
**Diagnosis**	No diagnosis problems detected<br>Model did not converge fully				
**Other information**	Dispersion parameter for Gaussian family taken to be 0.006940391<br><br>Null deviance: 0.64511 on 18 degrees of freedom<br>Residual deviance: 0.11799 on 17 degrees of freedom				

β = estimated coefficients for the fixed effect terms; SE = Standard Error; Intercept = AD-CON; Significance codes: *** 0.001; ** 0.01; * 0.05

Table S43: Summary of the selected model for analysis of muscle double chain/pro-Ctsb ratio of AD mice

**Method**	Generalized linear model (GLM)				
**Family distribution with link function**	Gaussian (identity)				
**Formula**	double chain/pro-Ctsb ratio ~ Treatment				
**Fixed effects**		**β**	**SE**	**t value**	**Pr(>|t|)**
	Intercept	0.8352	0.1440	5.801	2.13e-05***
	TreatCTSB	0.7712	0.1985	3.886	0.00119 **
**Diagnosis**	No diagnosis problems detected<br>Model did not converge fully				
**Other information**	Dispersion parameter for Gaussian family taken to be 0.1865633<br><br>Null deviance: 5.9892 on 18 degrees of freedom<br>Residual deviance: 3.1716 on 17 degrees of freedom				

β = estimated coefficients for the fixed effect terms; SE = Standard Error; Intercept = AD-CON; Significance codes: *** 0.001; ** 0.01; * 0.05
